# Environmental Enrofloxacin Exposure as a Modifiable Driver of Mitochondria‐Mediated Intestinal Aging and Barrier Dysfunction

**DOI:** 10.1111/acel.70526

**Published:** 2026-04-30

**Authors:** Kan Yu, Nengzheng Wang, Xinyi Huang, Yushu Qiu, Xuejian He, Xiaofeng Zhu, Xiaolan Zhou, Peng Yu, Gang Wei, Yan Pi, Ting Ni

**Affiliations:** ^1^ State Key Laboratory of Genetics and Development of Complex Phenotypes National Clinical Research Center for Aging and Medicine, Huashan Hospital, Collaborative Innovation Center of Genetics and Development, Human Phenome Institute, Center for Evolutionary Biology, Shanghai Engineering Research Center of Industrial Microorganisms, School of Life Sciences, Fudan University Shanghai China; ^2^ Department of Pediatric Infectious Diseases Xinhua Hospital, Shanghai Jiao Tong University School of Medicine Shanghai China; ^3^ State Key Laboratory of Reproductive Regulation and Breeding of Grassland Livestock Institutes of Biomedical Sciences, School of Life Sciences, Inner Mongolia University Hohhot China

**Keywords:** environmental enrofloxacin exposure, gut microbiota remodeling, intestinal mucosal barrier disruption, mitochondrial dysfunction, mitochondrial‐targeted therapy

## Abstract

Environmental antibiotic pollution is an underexplored contributor to gut aging and chronic intestinal diseases. We provide evidence that chronic exposure to enrofloxacin (ENR), a commonly detected veterinary antibiotic, accelerates gut aging and disease progression through a mitochondria‐centered mechanism. In a population‐based cross‐sectional analysis, recent antibiotic use was associated with increased biological age and a higher risk of diarrhea in middle‐aged and older adults, supporting a link between antibiotic exposure and impaired gut health and aging processes. Using zebrafish and intestinal epithelial cell models, we demonstrate that low‐dose ENR exposure impairs intestinal function, characterized by increased permeability, reduced mucus secretion, tight junction disruption, and chronic inflammation. Multi‐omics profiling revealed that ENR induced gut microbial dysbiosis, reduced metabolic diversity, and intestinal hypoxia. Mitochondrial dysfunction, particularly impaired oxidative phosphorylation, was identified as the key driver of epithelial damage. Remarkably, treatment with pyrroloquinoline quinone, a mitochondrial‐targeted antioxidant, reversed ENR‐induced mitochondrial injury, restored intestinal integrity, reduced inflammation, and partially normalized the microbiome. Stratified analyses in the human cohort showed that higher gut microbiota‐related diet quality and antioxidant capacity mitigated antibiotic‐associated aging and diarrhea risk. These findings highlight mitochondrial protection and microbiota optimization as promising therapeutic strategies.

## Graphical abstract

Environmentally relevant enrofloxacin accelerates intestinal aging by impairing epithelial mitochondrial function, disrupting barrier integrity, and reshaping the gut microbiota. Mitochondrial restoration with pyrroloquinoline quinone alleviates hypoxia, inflammation, and gut damage.

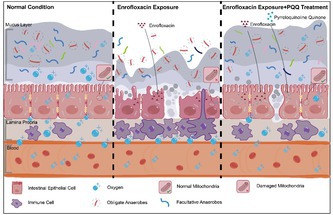

**Abbreviations**
ABX: antibiotic treatment
ADMET: absorption, distribution, metabolism, excretion, and toxicity
ANOVA: analysis of variance
BCA: bicinchoninic acid
BMI: body mass index
CDAI: dietary antioxidant index
CD‐HIT: cluster database at high identity with tolerance
CI: confidence interval
CRP: C‐reactive protein
CYC1: cytochrome c1
DAPI: 4′,6‐diamidino‐2‐phenylindole
DEGs: differentially expressed genes
DI‐GM: gut microbiota–related dietary index
DMEM: Dulbecco's modified Eagle medium
DMs: differentially abundant metabolites
DMSO: dimethyl sulfoxide
DNA: deoxyribonucleic acid
dpf: days post‐fertilization
ECL: enhanced chemiluminescence
ENR: enrofloxacin
FCCP: carbonyl cyanide‐p‐trifluoromethoxyphenylhydrazone
FDR: false discovery rate
FMT: fecal microbiota transplantation
GAPDH: glyceraldehyde 3‐phosphate dehydrogenase
GO: Gene Ontology
GSEA: gene set enrichment analysis
H&E: hematoxylin and eosin
HRP: horseradish peroxidase
IBD: inflammatory bowel disease
IEC‐6: intestinal epithelial cell line‐6
KD: equilibrium dissociation constant
KDM: Klemera–Doubal method
KEGG: Kyoto Encyclopedia of Genes and Genomes
LEfSe: linear discriminant analysis effect size
MANOVA: multivariate analysis of variance
MSigDB: Molecular Signatures Database
O2K: Oroboros Oxygraph‐2k high‐resolution respirometer
OMIM: Online Mendelian Inheritance in man
OR: odds ratio
OTU: operational taxonomic unit
PAS: periodic acid–Schiff
PBS: phosphate‐buffered saline
PCA: principal component analysis
PCoA: principal coordinates analysis
PCR: polymerase chain reaction
PGC‐1α: peroxisome proliferator–activated receptor gamma coactivator‐1α
PharmGKB: Pharmacogenomics Knowledgebase
PMSF: phenylmethylsulfonyl fluoride
PPI: protein–protein interaction
PQQ: pyrroloquinoline quinone
PVDF: polyvinylidene difluoride
qPCR: quantitative polymerase chain reaction
RIPA: radioimmunoprecipitation assay
ROS: reactive oxygen species
RRID: Research Resource Identifier
RT‐PCR: real‐time polymerase chain reaction
SDS‐PAGE: sodium dodecyl sulfate–polyacrylamide gel electrophoresis
SIRT1: sirtuin 1
SMILES: simplified molecular‐input line‐entry system
SPR: surface plasmon resonance
TBST: Tris‐buffered saline with Tween 20
TNF‐α: tumor necrosis factor‐α
WBC: white blood cell count

## 1 Introduction

Environmental factors are major extrinsic drivers of organismal aging (Argentieri et al. 2025; Huang et al. 2026; Patel et al. 2026). Recent evidence suggests that inflammaging may be context‐dependent and, in some populations, may be a byproduct of industrialized lifestyles rather than a universal feature of aging (Li et al. 2023; Franck et al. 2025). Pharmaceutical and personal care products are now widely recognized as emerging micro‐pollutants with ecological risks and potential bioactive effects at low concentrations (Ortuzar et al. 2022; Wilkinson et al. 2022), and antibiotics are an important subset given their widespread release and relevance to both ecological and human health risks (Larsson and Flach 2022; Hanna et al. 2023). Enrofloxacin (ENR) is a veterinary fluoroquinolone antibiotic (Bhatt and Chatterjee 2022; Zhang, Tang, et al. 2022) that is frequently detected in aquatic environments and can enter the food chain via animal production systems (Ben et al. 2022; Bhatt and Chatterjee 2022). Alarmingly high concentrations of ENR (e.g., μg/L levels in animal wastewater and high ng/L levels in rivers) have raised concerns regarding environmental contamination and long‐term exposure (Qiu et al. 2022; Wu et al. 2025). Although the European Union has limited ENR residues in meat products to a maximum of 100 μg/kg (Moga et al. 2021; Ben et al. 2022), concentrations in some regional surveys have been reported to exceed these limits (Wang et al. 2017). Human exposure occurs mainly through dietary intake and environmental background exposure, with antibiotics increasingly measurable using biomonitoring approaches (e.g., urinary detection) (Hu et al. 2022; Zhong et al. 2022), and population studies have reported non‐trivial prevalence of detectable antibiotic exposure in adults (Hu et al. 2022; Wang, Wang, et al. 2024). Together, these findings underscore the urgency of addressing long‐term, chronic exposure to environmental antibiotic residues and their implications for public health (Larsson and Flach 2022; Hanna et al. 2023).

Mounting evidence indicates that environmental factors—including medications and environmental pollutants—can contribute to intestinal aging by reshaping the gut microbiota and promoting chronic mucosal inflammation and barrier dysfunction (Ghosh et al. 2022). The intestine is increasingly recognized as a key target organ in organismal aging, with age‐onset barrier dysfunction linked to systemic health decline across model systems (Hohman and Osborne 2022). Intestinal aging is thought to be driven by converging processes including epithelial stress/senescence, barrier impairment, immune remodeling, and microbiome shifts (Li et al. 2023; Salazar et al. 2023). Senescence‐associated remodeling of the intestinal epithelium—often involving upregulation of canonical senescence markers such as CDKN1A/p21 and CDKN2A/p16, together with related cell‐cycle arrest programs—can reshape the local microenvironment and tissue function (Salazar et al. 2023; Ferreira‐Gonzalez et al. 2025). As these processes progress, mucosal barrier function becomes compromised (e.g., tight junction and mucus layer alterations), facilitating translocation of luminal contents and amplifying inflammation and permeability (Salazar et al. 2023; Neurath et al. 2025). Mitochondrial dysfunction is increasingly appreciated as an amplifier of epithelial stress responses and inflammatory signaling in the gut, with important implications for barrier integrity (Guerbette et al. 2022; Rath and Haller 2022). In parallel, aging is accompanied by gut microbiota dysbiosis, often characterized by reduced diversity and enrichment of pro‐inflammatory/opportunistic taxa that can promote low‐grade mucosal inflammation (Bosco and Noti 2021; Bradley and Haran 2024).

Antibiotics can influence intestinal health through multiple mechanisms, including microbiome disruption and direct effects on intestinal epithelial biology (Duan et al. 2022; Miller and Singer 2022). Recent syntheses highlight that antibiotics can compromise barrier function through both microbiota‐dependent and epithelial‐intrinsic pathways (Duan et al. 2022; Neurath et al. 2025). In addition, antibiotics can induce mitochondrial dysfunction and oxidative stress responses in host cells, with potential downstream impacts on epithelial homeostasis and inflammation (Miller and Singer 2022; Urbauer et al. 2024). However, the impact of chronic environmental (low‐dose, long‐term) antibiotic exposure on intestinal mitochondria and subsequent gut aging–related outcomes remains insufficiently defined (Bhatt and Chatterjee 2022; Hanna et al. 2023). Given the well‐established gut microbiota–epithelium–immune crosstalk in the pathogenesis of barrier dysfunction and chronic intestinal disease (Van Hul et al. 2024; Neurath et al. 2025), it is important to investigate how environmental ENR exposure perturbs these interconnected pathways.

In this study, we aimed to address three scientific questions: Does chronic antibiotic exposure associate with aging‐related traits and intestinal health at the population level (Argentieri et al. 2025)? What mechanisms link environmental ENR exposure to intestinal aging phenotypes (Li et al. 2023; Franck et al. 2025)? Based on the identified mechanism, is it feasible to therapeutically reverse or ameliorate antibiotic‐associated intestinal aging features (Rath and Haller 2022; Yan et al. 2024)? We combined population‐based analyses with zebrafish and cellular models to evaluate causal effects and mechanisms, and we further tested a mitochondria‐targeted intervention. These results address public health issues associated with antibiotic pollution and support mechanistically informed risk assessment and targeted mitigation strategies (Larsson and Flach 2022; Hanna et al. 2023).

## 2 Materials and Methods

### 2.1 Population Studies

We analyzed data from three NHANES cycles (2005–2006, 2007–2008, 2009–2010), including adults aged ≥ 20 years with complete information on medication use, aging biomarkers, bowel health, and covariates (Figure S11). Recent antibiotic exposure was defined using the 30‐day prescription medication files by identifying drugs classified as antibacterial agents; participants reporting at least one such drug were considered exposed. Outcomes included diarrhea, derived from the Bowel Health Questionnaire, and biological aging, estimated using the Klemera–Doubal method (KDM) to calculate biological age and age acceleration (residual of KDM age on chronological age). The Klemera‐Doubal method (KDM) biological age algorithm was applied to estimate individual biological age based on blood‐chemistry‐derived biomarkers (Klemera and Doubal 2006). The formula is as follows:

$$\mathrm{KDM}=\frac{\sum_{i=1}^{n}\left(x_{i}-q_{i}\right)\frac{k_{i}}{s_{i}^{2}}+\frac{\mathrm{CA}}{s_{\mathrm{BA}}^{2}}}{\sum_{i=1}^{n}{\left(\frac{k_{i}}{s_{i}}\right)}^{2}+\frac{1}{s_{\mathrm{BA}}^{2}}}$$

where *x*_*i*_ is the observed value of biomarker i; *q*_*i*_, *k*_*i*_, and *s*_*i*_ are the regression intercept, slope, and root mean squared error from regressing biomarker i on chronological age, respectively. *s*_BA_ is a scaling factor reflecting the variance in chronological age explained by the biomarker set, and CA refers to the chronological age.

Following prior studies, we selected ten biomarkers included in the NHANES dataset to calculate KDM: systolic blood pressure, albumin, alkaline phosphatase, blood urea nitrogen, creatinine, glycated hemoglobin, total cholesterol, lymphocyte percentage, white blood cell count, and mean cell volume (Huang et al. 2024). Participants with missing data on any biomarker required for KDM calculation were excluded from the KDM analyses (*n* = 572).

In a secondary analysis, we investigated whether different diet‐based strategies targeting the gut microbiota or antioxidant intake could modify antibiotic‐related outcomes using the dietary index for gut microbiota (DI‐GM) and the composite dietary antioxidant index (CDAI).

DI‐GM was calculated following previously published method (Wang, Xu, et al. 2024). Fourteen food groups or nutrients were included, yielding a total DI‐GM score ranging from 0 to 13, with higher scores reflecting more gut‐friendly dietary patterns. Detailed information on the components and scoring criteria for the DI‐GM is presented in Table S4.

CDAI was constructed based on intakes of multiple dietary antioxidants, including vitamins A, C, and E, β‐carotene, selenium, and zinc (Meng et al. 2024). Specifically, CDAI was defined as follows:

$$\text{CDAI}=\sum_{I=1}^{6}\frac{\left(\text{Individual intak}e_{i}-\mathrm{Mea}n_{i}\right)}{SE_{i}}$$

where Mean and SE denote the sex‐specific population mean and standard error, respectively. Higher CDAI scores indicate greater dietary antioxidant capacity.

Previous proteomic studies have shown that biological aging trajectories accelerate from approximately 45 years of age onward (Ding et al. 2025), so we restricted the analytic sample to adults aged > 45 years. Within strata defined by DI‐GM and CDAI, dichotomized at their median values, we then examined the associations between recent antibiotic use and KDM age acceleration and diarrhea (Li et al. 2025).

Covariates comprised sociodemographic factors (age, sex, race/ethnicity, marital status, income–poverty ratio), lifestyle factors (smoking, alcohol intake, total energy intake, BMI), and inflammatory indicators (C‐reactive protein [CRP], white blood cell count [WBC]). Survey‐weighted multivariable logistic regression was used to evaluate the associations between recent antibiotic use and diarrhea or aging acceleration, with results reported as adjusted odds ratios (95% confidence intervals). All analyses accounted for the complex NHANES sampling design, and two‐sided *p* < 0.05 was considered the threshold for statistical significance.

### 2.2 Experimental Design and Exposure Conditions

#### 2.2.1 Antibiotic Exposure

We selected ENR concentration (5000 ng/L) based on environmental monitoring studies reporting average ENR concentrations ranging from 978.8 to 34,400 ng/L in aquatic systems, including rivers and wastewater treatment effluents (Qiu et al. 2022). This concentration is environmentally relevant, reflecting realistic chronic exposure in the gut scenarios encountered by humans through water and dietary sources (Moga et al. 2021; Zhong et al. 2022). Exposure duration (30 days for zebrafish and 72 h for IEC‐6 (Intestinal Epithelial Cell line‐6, derived from rat small intestine crypt cells)) was chosen according to previously validated experimental models (Qiu et al. 2022) and preliminary experiments (Figure S1), ensuring physiological and toxicological relevance.

#### 2.2.2 Zebrafish Exposure and Husbandry

Enrofloxacin (ENR, purity > 99%, CAS No. 93106‐60‐6) was purchased from Yuanmu Biotechnology Co. Ltd. (Shanghai, China). Adult wild‐type AB zebrafish ( *Danio rerio* , RRID: ZFIN_ZDB‐GENO‐960809‐7, 120 dpf) were bred following previously described protocols (Yu et al. 2023). Zebrafish were housed under standard conditions (28°C ± 1°C; 14‐h light/10‐h dark cycle).

#### 2.2.3 Cell Culture Antibiotic Exposure

IEC‐6 cells (Intestinal Epithelial Cell line‐6, *Rattus norvegicus*, RRID:CVCL_0343) from the American Type Culture Center (Rockville, Maryland, USA) were maintained in DMEM supplemented with 10% fetal bovine serum, 1% sodium pyruvate, 0.1 U/mL bovine insulin, and 100 μg/mL penicillin/streptomycin at 37°C with 5% CO_2_. Cells (3 × 10^3^ cells/well) were seeded in 96‐well plates, incubated overnight, and then treated with ENR‐containing medium for 72 h, followed by PBS washing.

### 2.3 Fecal Microbiota Transplantation (FMT) Experiments

For FMT experiments, adult AB zebrafish microbiota were depleted using antibiotic‐supplemented feed for 14 days, as described previously (Liang et al. 2024). The antibiotic cocktail, as referred from previous studies, consisted of ampicillin (0.5 mg/kg), metronidazole (0.5 mg/kg), neomycin (0.5 mg/kg), and vancomycin (0.25 mg/kg). Feces from zebrafish (control or 5000 ng/L ENR‐exposed groups) were collected, freeze‐dried (−44°C, 6 Pa, 48 h), and added to fish diets (5% *w*/*w*). Zebrafish receiving fecal‐supplemented diets were assessed histopathologically after 30 days.

To assess bacterial colonization, fecal samples were collected from recipient zebrafish after antibiotic treatment (ABX) and at 30 days post‐FMT. Total bacterial DNA was extracted using the QIAamp Fast DNA Stool Mini Kit (Qiagen, Germany). Quantitative reverse transcription followed by polymerase chain reaction (qRT‐PCR) was performed to detect *Plesiomonas* and *Cetobacterium*, two representative genera identified by metagenomic sequencing as significantly altered in the ENR‐exposed group, and thus selected as indicators of colonization fidelity in FMT recipients (Rashidi et al. 2021). Successful colonization was inferred when the relative abundance trends of both *Plesiomonas* and *Cetobacterium* in the FMT recipient group mirrored those observed in their respective donor groups.

### 2.4 Pyrroloquinoline Quinone (PQQ) Treatment

PQQ (purity > 98%, CAS No. 72909‐34‐3; Shanghai Aladdin Biochemical Technology Co. Ltd.) was prepared as a stock solution at a concentration of 0.1 mg/mL. PQQ was supplemented into the zebrafish diet at a concentration of 0.1 μg/mg feed in the ENR‐exposed group. For cellular assays, IEC‐6 cells were co‐exposed to ENR (5000 ng/L) and PQQ (5000 μg/L) for 72 h. The selected PQQ concentrations were based on previous studies that demonstrated effective mitochondrial protection without cytotoxicity in both animal and cell models (Yan et al. 2024).

### 2.5 Intestinal Permeability and Histopathology

#### 2.5.1 Smurf Assay

The Smurf assay assessed intestinal integrity non‐invasively, as described previously (Sun et al. 2024). Zebrafish (150 dpf) received oral gavage of erioglaucine disodium salt. Intestinal permeability was determined after 15 min by observing blue discoloration. Ten biological replicates per group were performed.

#### 2.5.2 Intestinal Tissue Histology

Zebrafish intestines were dissected, fixed in 4% paraformaldehyde, dehydrated, and paraffin‐embedded according to prior methods (Kayani et al. 2022). Tissue sections (3 μm) underwent hematoxylin and eosin (H&E) staining and were examined microscopically (Nikon, Eclipse, TS100). Goblet cell numbers were quantified using Image‐Pro Plus (version 6.0; Media Cybernetics Inc.) with six biological replicates per group.

#### 2.5.3 Periodic Acid‐Schiff (PAS) Staining

PAS staining for intestinal mucus thickness was conducted as previously reported (Wang et al. 2023). Briefly, dissected intestinal tissues were gently cleared of mesentery, ligated, fixed in Carnoy's fixative (≤ 12 h), dehydrated, paraffin‐embedded, and stained using an AB‐PAS staining kit (Solarbio, G1285, Beijing, China) according to the manufacturer's instructions. Six biological replicates were included for each group in this assay.

#### 2.5.4 Immunofluorescent Staining

Intestinal tissues were fixed (4% paraformaldehyde, 15 min), blocked (10% goat serum albumin, 0.4% Triton X‐100), and incubated with primary antibodies from Servicebio: Cdkn1a (GB11153, 1:300), Cdkn2a (GB151143, 1:300), Tomm20 (GB151481, 1:1000), Grp75 (GB112273, 1:650), Cox5a (GB111676, 1:500), Hsp60 (GB112464, 1:800), Cox4 (GB11250, 1:200), CD3 (GB13014, 1:100), Mucin‐2 (GB11344, 1:500), Occludin (GB111401, 1:750), Zo‐1 (GB115686, 1:1000), and Claudin‐1 (GB112543, 1:1000). Sections were then incubated with Cy3‐labeled goat anti‐rabbit IgG (H + L) secondary antibody (GB21303, Servicebio; 1:300) and counterstained with DAPI (Solarbio, C0065; 10 μg/mL). Images were obtained via confocal microscopy (Nikon A1). For each experimental group, immunofluorescence staining was performed on intestinal samples from six biologically independent adult zebrafish, with each sample derived from a distinct individual. Fluorescence intensities of target proteins were quantified using ImageJ software, and relative density was determined by calculating the ratio of integrated fluorescence intensity of each target protein to that of DAPI within the same region of interest.

#### 2.5.5 Hypoxia Marker Staining

Hypoxia‐sensitive dye (Image‐iT Green Hypoxia Reagent, I14834; Thermo Fisher Scientific) was applied to fixed tissues at a working concentration of 5 μM for 2 h. Subsequent immunostaining followed as described above, with imaging performed by Nikon A1 confocal microscopy. Each experimental group included six biologically independent intestinal samples, with each sample obtained from a separate adult zebrafish. Fluorescence intensities of target proteins were quantified using ImageJ software, and relative density was determined by calculating the ratio of integrated fluorescence intensity of each target protein to that of DAPI within the same region of interest.

### 2.6 Mitochondrial Respiratory Measurements

IEC‐6 cell respiration was measured with high‐resolution respirometry (Oxygraph‐2K; Oroboros Instruments, Innsbruck, Austria). Oxygen flux (pmol·s^−1^·mL^−1^) was recorded in real time. Cells suspended in DMEM underwent sequential titrations of ENR (500, 1000, 5000 ng/L) followed by oligomycin (2 μg/mL), carbonyl cyanide‐p‐trifluoromethoxyphenylhydrazone (FCCP), rotenone, and antimycin A. Dimethyl sulfoxide (DMSO) served as a solvent control. Each experimental condition was independently assessed in three biological replicates.

### 2.7 RNA Extraction and qRT‐PCR

Zebrafish intestinal tissues and IEC‐6 cell supernatants underwent RNA extraction using Eastep RNA Kit (Promega) and RNeasy Mini Kit (Qiagen), respectively. For zebrafish, intestinal tissues from three individual fish were pooled to generate one sample, and three biological replicates were prepared per treatment group. cDNA synthesis utilized Prime‐Script RT Reagent Kit (Takara Bio). qRT‐PCR was performed with Eastep qPCR Master Mix (Promega) on an ABI StepOne Plus System (Applied Biosystems). Gene expression levels in zebrafish samples were normalized to β‐actin, while those in IEC‐6 cells were normalized to GAPDH. Relative expression was calculated using the 2^−ΔΔCt^ method, and all reactions were performed in triplicate. Primer sequences used for qPCR are listed in Table S5.

### 2.8 Western Blot Analysis

Protein extracts derived from zebrafish intestinal tissues or IEC‐6 cells (lysed in RIPA buffer containing PMSF) were subjected to BCA quantification. For each zebrafish treatment group, intestines from three individual fish were pooled as one sample, and three biological replicates were prepared. Similarly, cell experiments were conducted with three biological replicates per condition. Proteins separated via SDS‐PAGE were transferred to PVDF membranes. Membranes were blocked (5% skim milk, TBST), incubated overnight with primary antibodies (TNF‐α [KPAB0373, AssayGenie], Tomm20 [GB111481, Servicebio], GAPDH [GB15002, Servicebio], 1:2000), then incubated with HRP‐conjugated secondary antibodies (GB23303, 1:2000; Servicebio). Signals were visualized using ECL substrate (Thermo, 32106) and quantified using ImageJ, normalized to GAPDH.

### 2.9 Omics Analyses

#### 2.9.1 Metabolomics

Intestinal tissue samples from six adult zebrafish at 150 dpf per group were pooled as a composite sample (three biological replicates for each treatment) and subjected to metabolomic detection and analysis using previously described methods (Yu et al. 2024). Briefly, after the samples were freeze‐dried, tissue extract was added at a ratio of 100 mg/mL and then ground. Subsequently, after sonication at 25°C and centrifugation at 5000× *g*, the supernatant was filtered and analyzed using ultra‐performance liquid chromatography–tandem mass spectrometry (Thermo, MA, USA). An electrospray ionization injector was used for mass spectrometric analysis, where parameters such as spray voltage, scan range, capillary temperature, and resolution were set according to previous experiments (Yu et al. 2024). Finally, the obtained data were converted into the mzXML format, and peak identification, filtering, and alignment were performed using the R XCMS software package (v4.2.0). To assess the statistical significance of sample clustering observed in the Principal Component Analysis (PCA) of metabolomic profiles, we performed multivariate analysis of variance (MANOVA) based on the first two principal components. The differentially abundant metabolites (DMs) between different groups of samples satisfied |log2 FC| ≥ 1 and *p*‐value ≤ 0.05. KEGG pathway enrichment analysis of the DMs was performed by annotating metabolites to KEGG compound identifiers and mapping them to KEGG pathways using the Kyoto Encyclopedia of Genes and Genomes database (https://www.kegg.jp/); enriched pathways were then identified by over‐representation analysis and retained for interpretation when *p*‐value ≤ 0.05.

#### 2.9.2 Metagenomic Sequencing

Metagenomic sequencing was performed as previously described (Yu et al. 2024). Each sample consisted of pooled fecal material collected from ten adult zebrafish, with three biological replicates. Briefly, after collecting feces, metagenomic DNA was extracted using the Qiagen PowerFecal kit (Qiagen). The Nextera DNA Flex library preparation kit (Illumina, California, USA) was used to create metagenomic libraries and metagenomic sequencing was performed using an Illumina Hiseq X Ten (Illumina) sequencer. The quality of the raw sequencing data was evaluated using Fastp (v0.36). After metagenome assembly using MEGAHIT (v1.2.9) and metaSPAdes (v3.13), open reading frames (ORFs) were predicted using Prodigal (v2.60), and redundant predicted genes were removed using CD‐HIT (v2.60). DIAMOND (version 0.8.20) was used to compare the gene set with databases, such as KEGG and Gene Ontology (GO), to obtain the species and functional annotation information of the genes. GraPhlAn (http://segatalab.cibio.unitn.it/tools/graphlan) was used to construct a taxonomic tree of intestinal microbiota. To assess the statistical significance of sample clustering observed in the PCA of metagenomic profiles, we performed MANOVA based on the first two principal components. The α‐diversity indices, including Shannon, Simpson, and Chao1 indices, were calculated using QIIME2 (version 2022.2). The association of metadata with the gut microbiota and metabolomic parameters was analyzed using Spearman's rank correlation test. To ensure taxonomic reliability, microbial species with relative abundance below 0.01% across all samples were excluded from downstream analysis (Blanco‐Miguez et al. 2023).

#### 2.9.3 16S rRNA Sequencing

Zebrafish fecal DNA (30‐day ENR exposure) underwent PCR amplification. Each sample consisted of pooled fecal material collected from ten adult zebrafish, with three biological replicates. MiSeq sequencing (Illumina) was performed, followed by analyses including OTU clustering, diversity analysis, and Principal Coordinates Analysis (PCoA; QIIME, Majorbio). To assess the statistical significance of sample clustering observed in the PCoA, we performed MANOVA based on the first two principal components. For 16S rRNA‐based taxonomic profiling, microbial genera with a relative abundance below 0.01% across all samples were excluded prior to statistical analysis to minimize the impact of rare taxa and sequencing noise (Blanco‐Miguez et al. 2023).

#### 2.9.4 Statistical Analysis of Gut Microbial Abundance

Linear discriminant analysis Effect Size (LEfSe) was utilized to identify taxa significantly enriched between experimental groups. LEfSe analyses were conducted using the Galaxy platform (http://huttenhower.sph.harvard.edu/galaxy/) with default parameters (*α*‐value = 0.05, logarithmic LDA score threshold = 2.0). To evaluate the impact of fluoroquinolone antibiotic exposure on gut microbial abundance in zebrafish, we first assessed the normality of genus‐level abundance data in both control and antibiotic‐exposed groups. Metagenomic sequencing data from untreated zebrafish fecal samples (*n* = 8, obtained from PRJNA806371, SRR12456161, SRR12456162, and SRR12456170) and zebrafish fecal samples exposed to fluoroquinolone antibiotics (levofloxacin at 5000 ng/L, pharmacologically similar to enrofloxacin, and enrofloxacin exposure, *n* = 6) were analyzed. Shapiro–Wilk tests indicated that abundance data of the genera *Cetobacterium* and *Plesiomonas* in both the untreated and antibiotic‐exposed groups conformed to normal distributions (*p* > 0.05), thus justifying the subsequent use of independent‐samples *t*‐tests for between‐group comparisons. For the taxonomic tree visualizations in Figures 4B and 11I, each node was presented as a taxon‐centered pie chart. Reads assigned to a given taxon were first summed across biological replicates within each compared group, and the relative contribution of each group to that taxon was then calculated from these pooled reads. Thus, the colored sectors represent the contribution of each group to the indicated taxon, while the percentage shown next to each taxon indicates its overall relative abundance in the corresponding dataset/panel. These pie charts were intended to show the distribution of a given taxon across groups, rather than the overall taxonomic composition of a single condition.

#### 2.9.5 RNA Sequencing

The RNeasy Mini Kit (Qiagen, Hilden, Germany) was used to extract total RNA and the PrimeScript RT Kit (Takara Bio, Shiga, Japan) was used to create a cDNA library. For each experimental group, three biological replicates were prepared, with total RNA extracted independently from each replicate. Sequencing libraries were generated using VAHTSTM mRNA‐seq V2 Library Prep Kit for Illumina following manufacturer's recommendations and index codes were added to attribute sequences to each sample. High‐throughput sequencing was performed on multiple samples using the paired‐end sequencing mode on the Illumina HiSeq sequencing platform (Illumina, California, USA). DESeq2 software (v1.16.1) was used to screen for differentially expressed genes (DEGs) between samples from different groups (meeting |log_2_ FC| ≥ 1 and *p*‐value ≤ 0.05). Gene set enrichment analysis (GSEA) was performed using GSEA software (v4.x) with hallmark gene sets from MSigDB. Gene expression data was preranked by signal‐to‐noise ratio, and 1000 permutations were conducted. Gene sets with nominal *p* < 0.05 and FDR < 0.25 were considered significantly enriched. Functional analysis of GO terms was performed using the TopGO package (v2.58.0).

### 2.10 Network Toxicological Analyses

SMILES structures of antibiotics obtained from PubChem were analyzed using ADMETlab 2.0 and SwissADME. Predicted targets from SwissTargetPrediction, ChEMBL, PharmMapper, and UniProt were integrated, and the aging‐related targets were obtained in GeneCards, Online Mendelian Inheritance in Man (OMIM), and PharmGKB.

### 2.11 Prediction and Identification of ENR Targets

#### 2.11.1 Molecular Docking

ENR and protein receptor structures (UniProt, PubChem) were docked (AutoDock 4). Binding energies and visualization (Discovery Studio 2019) were recorded.

#### 2.11.2 Protein Sequence Identity

Sequence identity among human, rat, and zebrafish proteins (UniProt, NCBI) was calculated using Jalview (version 2.11.4.1).

#### 2.11.3 Surface Plasmon Resonance (SPR)

SPR was determined using a Biacore 8K instrument (GE Healthcare, Uppsala, Sweden). The recombinant Cytochrome c1 (CYC1) protein (P2142, Fine Test) was immobilized on a sensor chip using the amine‐coupling method according to standard protocols. CYC1 protein was diluted in sodium acetate buffer, pH 4.5. Various concentrations of ENR were subsequently injected as analytes. To estimate the affinity, the binding assay was examined at 25°C at a flow rate of 30 μL/min using PBS buffer. The affinity constants of binding were obtained using the 1:1 Langmuir binding model via BIA evaluation software.

### 2.12 Statistical Analysis

All statistical analyses were performed using GraphPad Prism 8 and R (version 4.4.0). Statistical methods for NHANES, metabolomic, metagenomic, 16S rRNA, and RNA‐seq analyses are described in the corresponding subsections above. For other experimental data, comparisons among more than two groups were performed using one‐way ANOVA followed by Tukey's multiple‐comparisons test when all pairwise comparisons were of interest, or Dunnett's post hoc test when comparisons were made only against a prespecified control or reference group. Comparisons between two groups were conducted using two‐tailed Student's *t*‐test. Categorical variables were analyzed using the chi‐squared test. For analyses involving multiple testing, *p*‐values were adjusted using the Benjamini–Hochberg method. Unless otherwise indicated, a two‐sided *p*‐value < 0.05 was considered statistically significant.

## 3 Results

### 3.1 Clinically Prescribed Antibiotic Use Is Associated With Aging in the Population

**FIGURE 1:**
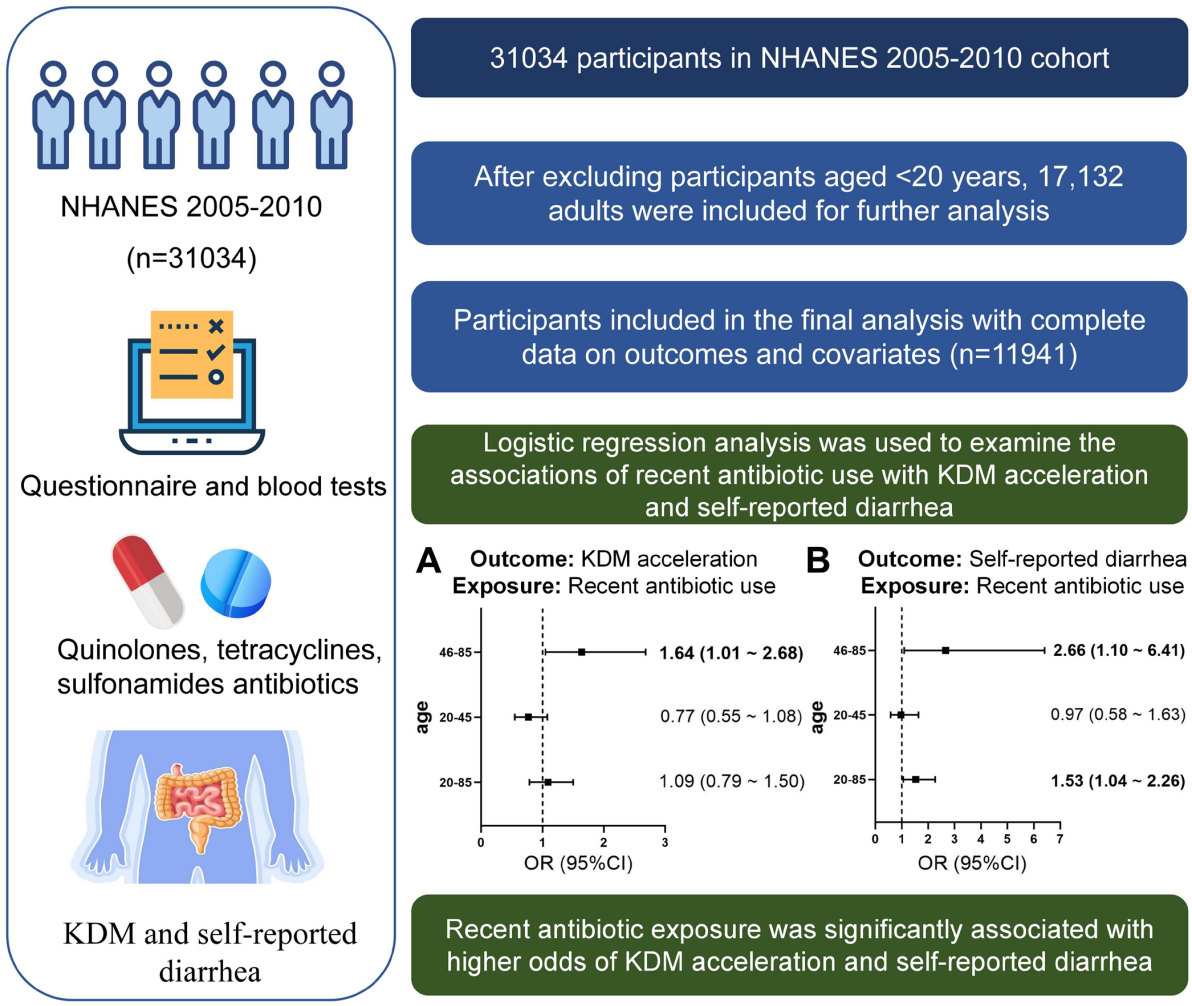
Population‐based analysis of associations of antibiotic exposure with KDM acceleration and self‐reported diarrhea. The schematic on the left summarizes the data source and key variables. The flow chart on the right shows sample selection. Survey‐weighted logistic regression analysis was used to examine the associations of recent antibiotic use with KDM acceleration (A) and self‐reported diarrhea (B) across age strata. Forest plots display adjusted ORs and 95% CIs. CI, confidence intervals; KDM, Klemera–Doubal biological age; OR, odds ratio.

Our analysis revealed that among individuals aged 46 years and older, increased Klemera–Doubal method (KDM) biological age and recent diarrhea were both significantly associated with antibiotic use within the past month (Figure 1, Table S2). Specifically, recent antibiotic use was significantly associated with higher odds of KDM acceleration (OR = 1.64, 95% CI: 1.01–2.68) and self‐reported diarrhea (OR = 2.66, 95% CI: 1.10–6.41). These results suggest that antibiotic use in older adults is associated with accelerated biological aging and an increased likelihood of diarrhea, highlighting the susceptibility of individuals over 45 years of age to antibiotic‐related adverse outcomes.

Considering that the observed phenotypes (increased KDM and diarrhea) could potentially be caused by infectious diseases rather than reflecting an effect of antibiotic use per se, we further adjusted for inflammatory markers indicative of infection (white blood cell count and C‐reactive protein [CRP]) in Model 2. After adjusting for these inflammatory markers, the associations of KDM and diarrhea with antibiotic use remained statistically significant (Table S3). These findings suggest that antibiotic use may be independently associated with increased biological aging and impaired gut function, beyond the influence of infection status. Although these observational findings reflect recent clinically prescribed antibiotic use rather than chronic environmental antibiotic exposure, they provide population‐level, hypothesis‐generating evidence that antibiotic exposure is linked to biological aging and impaired gut function, thereby motivating our subsequent hypothesis that chronic low‐dose environmental antibiotic exposure may likewise contribute to aging‐associated intestinal dysfunction.

### 3.2 ENR Exposure Impairs Gut Barrier Integrity via Mucus Reduction and Tight Junction Disruption

**FIGURE 2:**
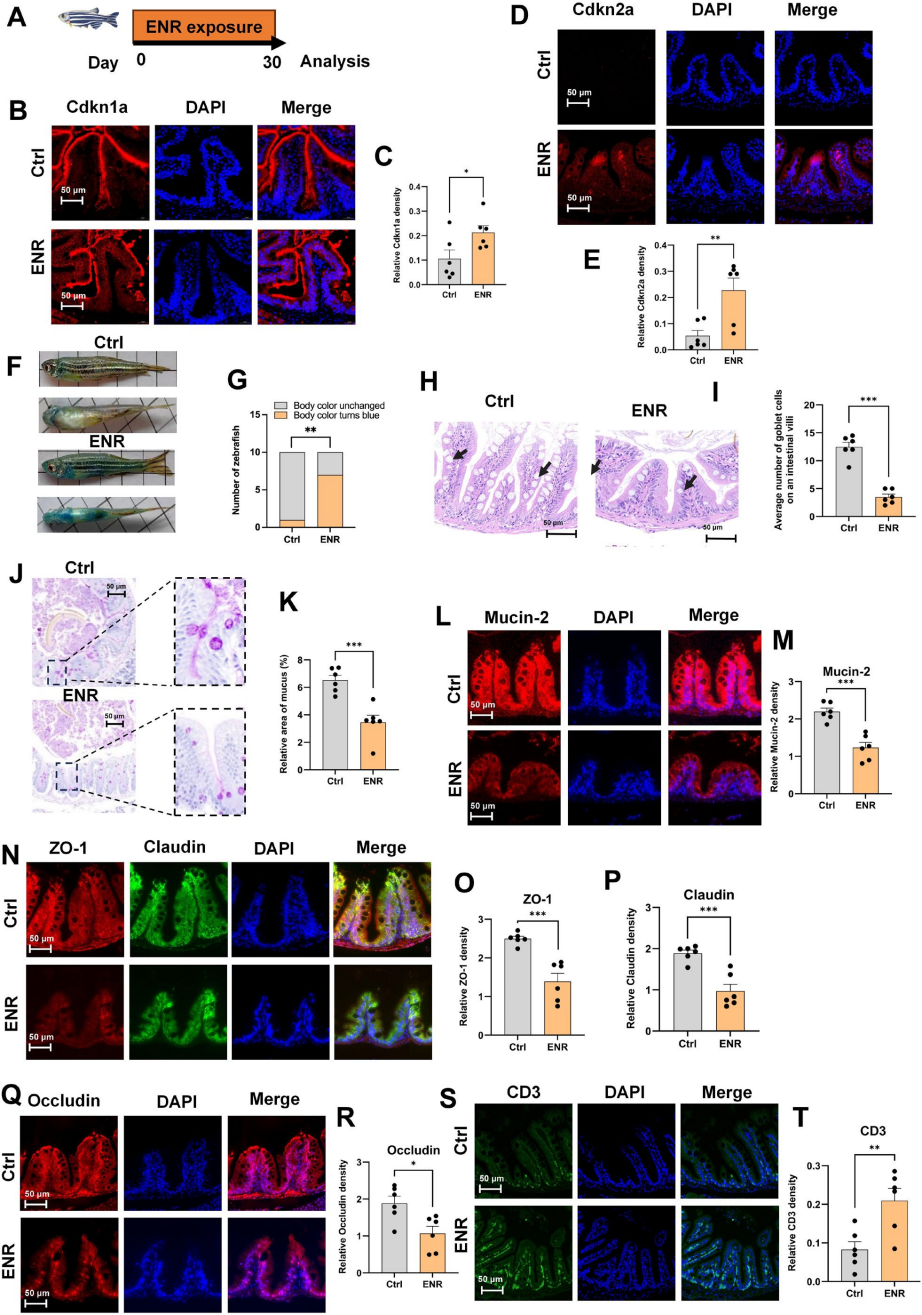
Effects of ENR exposure on intestinal barrier integrity in zebrafish. (A) Experimental design illustrating the exposure protocol. (B, C) Representative immunofluorescence images of Cdkn1a and quantification. (D, E) Representative immunofluorescence images of Cdkn2a and quantification. (F, G) Zebrafish intestinal permeability assessment using Smurf assay (*n* = 10). (H) Hematoxylin and eosin staining of zebrafish intestinal tissues. (I) Quantification of goblet cells in zebrafish intestinal tissues. (J, K) Representative Periodic Acid‐Schiff (PAS) staining and quantification of mucus production in zebrafish intestinal tissue. (L–R) Representative immunofluorescence images of intestinal tight junction proteins (Occludin, Mucin‐2, Zo‐1, Claudin) and quantification. (S, T) Representative immunofluorescence images of intestinal CD3‐positive T cells and quantification. Data are presented as the mean ± standard error of the mean. Statistical significance was assessed using Student's *t*‐test. *p < 0.05, **p < 0.01, ***p < 0.001.

To further validate whether the association observed in population studies between antibiotic use, biological aging, and gut dysfunction has potential causality, and to explore the possible biological mechanisms underlying this relationship, we designed animal experiments to simulate the direct impact of environmental‐level antibiotic exposure on gut health. We selected an ENR exposure concentration of 5000 ng/L based on previous literature (Qiu et al. 2022; Tian et al. 2023) and preliminary epithelial cell experiments (Figure S1). After 30 days of ENR exposure, the expression levels of both Cdkn1a and Cdkn2a in the intestinal tissue of adult zebrafish were significantly elevated, suggesting that ENR exposure induces an intestinal aging phenotype (Figure 2A–E). To determine whether chronic low‐dose ENR exposure impairs aging‐related intestinal barrier integrity, we performed a Smurf assay and found a significantly higher proportion of ENR‐exposed zebrafish with increased intestinal permeability than controls (Figure 2F,G).

The number of goblet cells—which secrete Mucin‐2, a primary mucus component—was significantly reduced by 65% compared to the control group (Figure 2H,I). Correspondingly, PAS staining further demonstrated a substantial reduction in intestinal mucus area in ENR‐treated fish (Figure 2J,K). Intestinal mucus is an essential component of the chemical barrier (Leonardi et al. 2022). Consistently, Mucin‐2 expression was also markedly decreased (Figure 2L,M), indicating impairment of intestinal mucosal function (Nystrom et al. 2021). Since tight junction proteins play a crucial role in maintaining intestinal mechanical integrity (Zhang et al. 2024), we next assessed the expression of Occludin, Zo‐1, and Claudin via immunofluorescence. The results showed that ENR exposure significantly reduced the expression of these proteins in intestinal epithelial cells (Figure 2N–R). Additionally, immunofluorescence staining revealed a marked increase in intestinal CD3‐positive T cells following ENR exposure (Figure 2S,T), likely reflecting inflammation associated with compromised intestinal barrier integrity (Mehandru and Colombel 2021). These results suggest that ENR exposure impairs intestinal mucosal barrier function in zebrafish.

### 3.3 ENR Exposure Remodels Gut Microbial Composition and Host Metabolism

**FIGURE 3:**
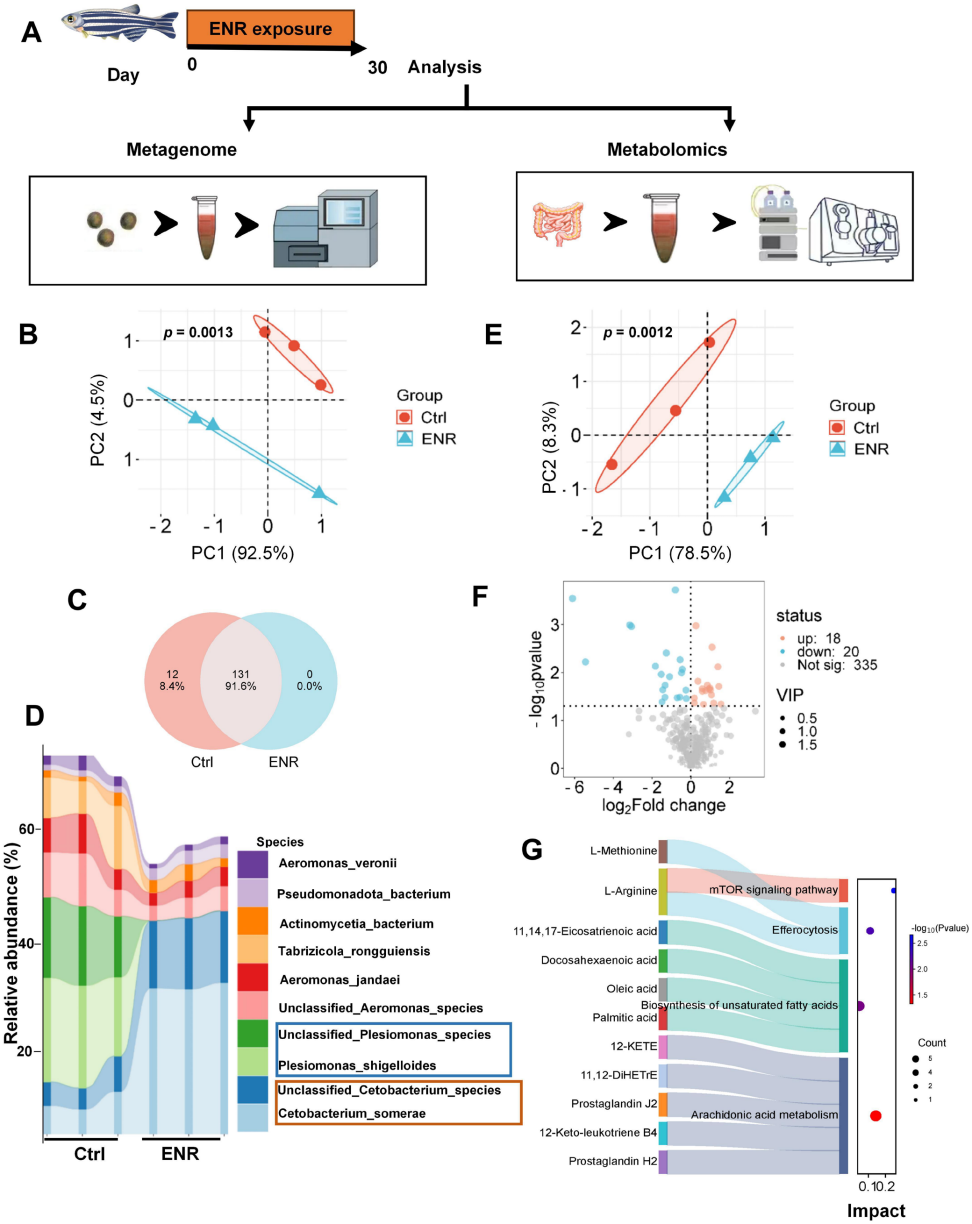
Impact of ENR exposure on gut microbiota and metabolites in zebrafish. (A) Overview of the experimental approach used for microbiome and metabolomic analyses. (B) Principal Component Analysis (PCA) illustrating differences in gut microbiota composition at the species level between control and ENR‐exposed groups. (C) Venn diagram showing shared and unique microbial species in control and ENR‐exposed zebrafish. (D) Relative abundance and composition of gut microbiota at the species level. (E) PCA plot depicting differences in gut tissue metabolites between the control and ENR‐treated groups. (F) Volcano plot identifying significantly altered metabolites between groups, with highlighted metabolites exhibiting significant fold changes. (G) Sankey diagram illustrating associations between significantly altered metabolites and related metabolic pathways (KEGG pathways).

Impairment of intestinal barrier function often coincides with changes in gut microbiota composition and metabolic profiles (Shi et al. 2017). To investigate whether ENR‐induced intestinal barrier dysfunction is mediated through changes in gut microbiota, we collected fecal and intestinal tissue samples from zebrafish exposed to ENR for 30 days and performed untargeted metagenomic and metabolomic analyses (Figure 3A).

PCA demonstrated distinct clustering of samples, with the first two principal components explaining 97% and 86% of the total variance for microbiome and metabolites, respectively (Figure 3B,E). Venn diagram analysis of bacterial species revealed shifts in microbiota composition following ENR exposure, with 8.4% of original bacterial species lost (Figure 3C). Among the top ten most abundant bacterial species, the relative abundance of facultative anaerobes decreased after ENR exposure (Figure 3D). Additionally, the metabolomic volcano plot identified 20 significantly upregulated and 18 significantly downregulated metabolites upon ENR treatment (Figure 3F). Kyoto Encyclopedia of Genes and Genomes (KEGG) pathway analysis further highlighted significant alterations in amino acid metabolism pathways, including efferocytosis, unsaturated fatty acid biosynthesis, and arachidonic acid metabolism (Figure 3G). These findings indicate that ENR exposure substantially reshapes intestinal microbial community structure and metabolic networks.

**FIGURE 4:**
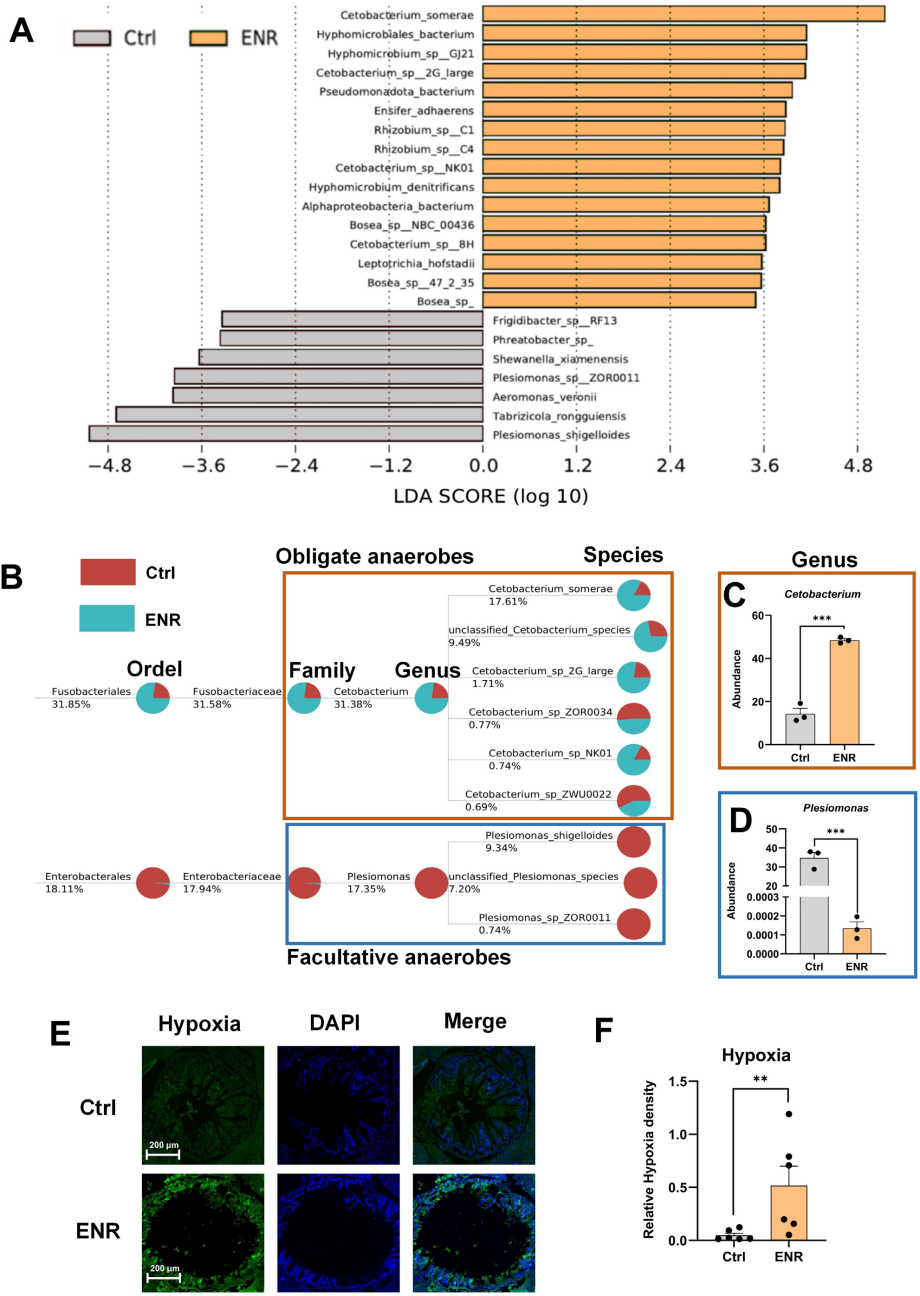
Impact of ENR exposure on intestinal hypoxia and microbiota structure in zebrafish. (A) Linear discriminant analysis (LEfSe) highlighting the most significantly altered bacterial species between control and ENR‐exposed groups. (B) Taxonomic classification of selected bacterial taxa, including *Cetobacterium somerae* and *Plesiomonas shigelloides* , across hierarchical levels (order, family, genus, and species). The percentage shown next to each taxon indicates its overall relative abundance in the corresponding dataset. Each taxonomic node is displayed as a pie chart, in which the colored sectors represent the contribution of the compared groups to that taxon. For each taxon, reads assigned to that taxon were first summed across biological replicates within the same treatment group, and the group‐level proportion was then calculated based on these pooled reads. (C, D) Quantitative changes in the relative abundances of *Cetobacterium somerae* (C) and *Plesiomonas shigelloides* (D) following ENR exposure. (E, F) Immunofluorescence staining for intestinal hypoxia markers and quantitative analysis. Data are presented as the mean ± standard error of the mean. Statistical significance was assessed using Student's *t*‐test. *p < 0.05, **p < 0.01, ***p < 0.001.

Further analysis using Linear discriminant analysis Effect Size (LEfSe) identified the top 50 significantly altered bacterial species, with *Cetobacterium somerae* and *Plesiomonas shigelloides* exhibiting the most prominent changes (Figure 4A). Taxonomic tracing at the order, family, and genus levels, along with statistical analysis, demonstrated a significant increase in the abundance of obligate anaerobe *Cetobacterium* and a significant decrease in the facultative anaerobe *Plesiomonas* following ENR exposure (Figure 4B–D). In zebrafish, core gut microbiota such as the obligate anaerobe *Cetobacterium* and facultative anaerobe *Plesiomonas* have been shown to significantly shift under chronic hypoxic conditions (Basak and Chakraborty 2024). To confirm whether these microbial changes correlate with intestinal hypoxia, pathological staining revealed that intestinal hypoxia levels increased significantly in ENR‐exposed zebrafish compared to controls (Figure 4E,F). These findings provide evidence that ENR exposure disrupts the intestinal microbiome and metabolic profiles.

### 3.4 ENR‐Induced Microbiota Dysbiosis Promotes Intestinal Inflammation Without Compromising Gut Barrier Integrity

**FIGURE 5:**
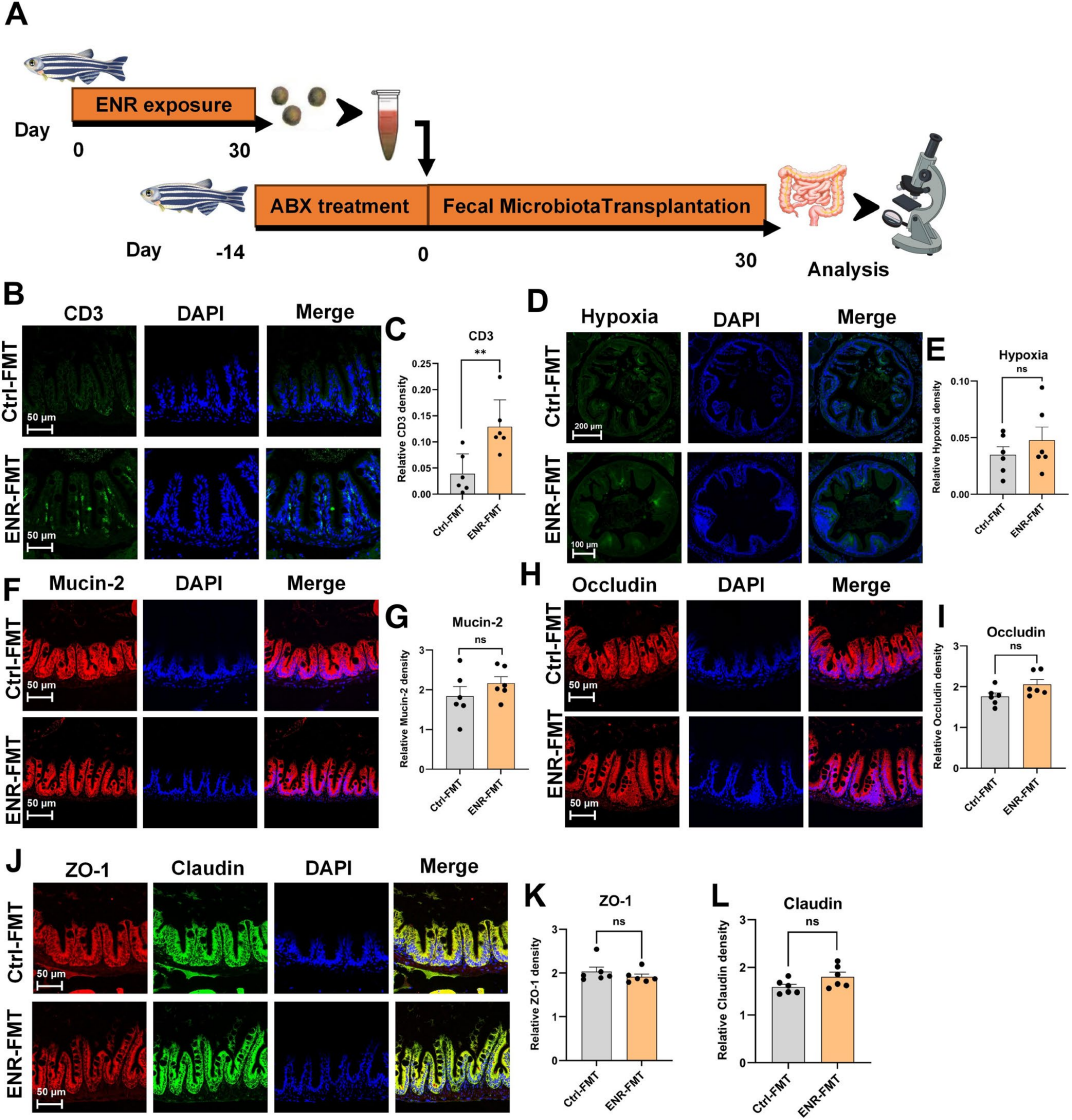
Effects of fecal microbiota transplantation (FMT) on intestinal barrier function. (A) Experimental overview illustrating the procedure of FMT following antibiotic (ABX) treatment and ENR exposure. (B, C) Representative immunofluorescence images of intestinal CD3‐positive T cells and quantification after FMT. (D, E) Representative immunofluorescence images of hypoxia markers and quantification after FMT. (F–L) Representative immunofluorescence images of intestinal tight junction proteins (Mucin‐2, Occludin, Zo‐1, Claudin) and quantification after FMT. Data are presented as the mean ± standard error of the mean. Statistical significance was assessed using Student's *t*‐test. *p < 0.05, **p < 0.01, ***p < 0.001.

To investigate whether ENR‐induced intestinal barrier damage and inflammation are mediated by alterations in the gut microbiome, fecal microbiota from ENR‐exposed zebrafish were transplanted into antibiotic treatment (ABX) recipient zebrafish (Figure 5A). To confirm successful colonization following FMT, we first assessed the relative abundance of *Cetobacterium* and *Plesiomonas*, two representative bacterial species previously identified in metagenomic analysis. Their abundance patterns in recipients mirrored those of ENR‐exposed donors, indicating microbiota engraftment (Figure S5B–D). Immunofluorescence analysis demonstrated a significant increase in CD3‐positive T‐cells in the intestinal lymphatic layer after FMT from ENR‐exposed donors, indicating microbiota‐driven intestinal inflammation (Figure 5B,C). However, intestinal hypoxia levels and expression of tight junction proteins showed no significant changes following transplantation (Figure 5D–L). These results suggest that while ENR‐altered microbiota contribute to intestinal inflammation, they alone are insufficient to cause direct impairment of intestinal barrier integrity or increased hypoxia.

To further clarify how microbiota changes might promote intestinal inflammation, we analyzed the relationship between gut microbiota composition and intestinal metabolites. Analysis of intestinal metabolites showed significant increases in pro‐inflammatory metabolites (11,12‐DiHETrE, 12‐Keto‐leukotriene B4, palmitic acid, asymmetric dimethylarginine, 11,14,17‐Eicosatrienoic acid) (Jin et al. 2010; Mao et al. 2017; Sjodin et al. 2018; Castoldi et al. 2021; Ermis et al. 2024) and reductions in anti‐inflammatory metabolites (L‐Methionine, prostaglandin J2, docosahexaenoic acid, 5′‐methylthioadenosine, oleic acid, dehydroepiandrosterone, L‐Arginine, perillyl alcohol) (Repa et al. 2004; Tabbaa et al. 2013; Rutkowski et al. 2014; Paredes and Andarawis‐Puri 2016; Puppala et al. 2022; Zhang, Zeng, et al. 2022; Gao et al. 2024; Luo et al. 2025) after ENR exposure (Figure S4C,D). These findings suggest that ENR‐induced microbiota dysbiosis may promote intestinal inflammation by altering intestinal metabolic profiles.

The gut microbiome influences host metabolism, so we analyzed the abundance of microbial genes involved in amino acid, lipid, energy, and carbohydrate metabolism. The results revealed that ENR exposure broadly reduced the abundance of microbial genes associated with these metabolic pathways (Figures S2F,G, S3A,B, and S4A,B), potentially due to intestinal hypoxia (Zong et al. 2024). Correlation analysis further indicated that both facultative and obligate anaerobic bacteria were associated with elevated pro‐inflammatory metabolites and decreased anti‐inflammatory metabolites, linking microbiome‐driven metabolic changes directly to intestinal inflammation (*p* < 0.05, |β| > 0.9) (Figures S3C and S4E). These findings confirm that while microbiota changes may promote inflammation, they do not directly impair the intestinal barrier, highlighting the complexity of the interaction between microbiome, metabolism, and gut health.

### 3.5 Mitochondrial Dysfunction in Epithelial Cells Mediates ENR‐Induced Intestinal Damage

**FIGURE 6:**
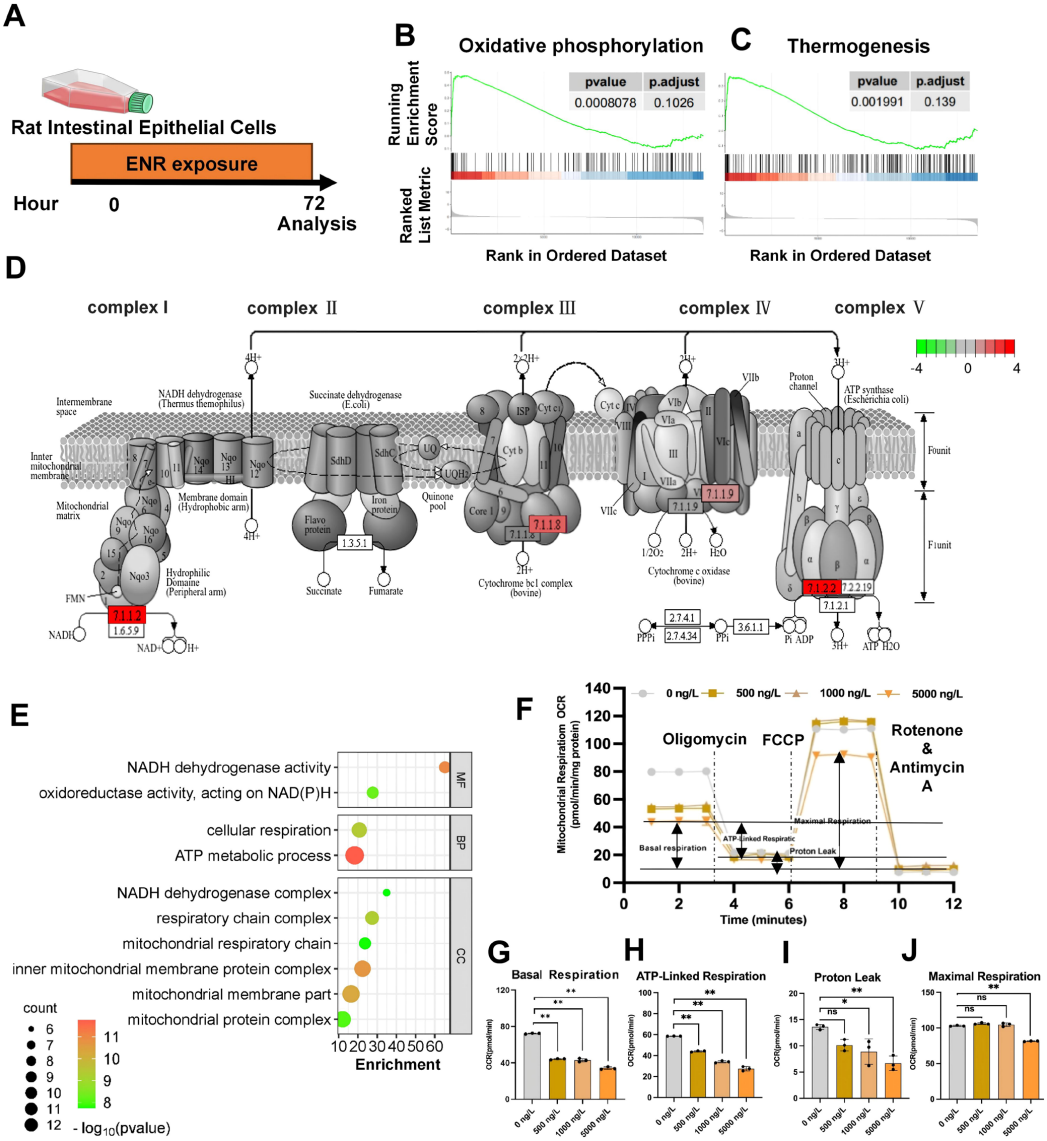
Impact of ENR exposure on zebrafish intestinal epithelial cells. (A) Overview of experimental procedures used to examine intestinal epithelial responses after ENR exposure. (B, C) Gene Set Enrichment Analysis (GSEA) plots highlighting enrichment in oxidative phosphorylation (B) and thermogenesis pathways (C). (D) Products of genes with significant changes in the oxidative phosphorylation pathway. (E) Gene Ontology (GO) enrichment analysis of mitochondrial function‐related pathways significantly impacted by ENR exposure. (F) Mitochondrial oxygen consumption rate (OCR) was recorded at baseline and after the sequential injection of oligomycin, FCCP, and a mixture of rotenone and antimycin A. (G, J) Quantitative analyses of mitochondrial respiratory parameters: Basal respiration (G), ATP‐linked respiration (H), proton leak (I), and maximal respiration (J). Data are presented as the mean ± standard error of the mean. Statistical significance was assessed using one‐way ANOVA. *p < 0.05, **p < 0.01, ***p < 0.001.

To explore the underlying causes of ENR‐induced intestinal hypoxia and barrier damage, we exposed IEC‐6 cells to 5000 ng/L ENR for 72 h and performed RNA sequencing analysis (Figure 6A). Gene set enrichment analysis (GSEA) showed that oxidative phosphorylation and thermogenesis pathways were significantly upregulated (Figure 6B,C). Most genes encoding mitochondrial complex subunits in the oxidative phosphorylation pathway were significantly upregulated (Figure 6D). Consistent with this, Gene Ontology (GO) analysis revealed significant enrichment of terms related to energy metabolism and mitochondrial functions following ENR exposure (Figure 6E).

Next, we directly assessed mitochondrial respiratory function in IEC‐6 cells using the Oroboros Oxygraph‐2k high‐resolution respirometer (O2K) (Figure 6F). ENR significantly impaired basal respiration, ATP‐linked respiration, proton leak, and maximal respiration in IEC‐6 cells, exhibiting clear concentration‐dependent effects (Figure 6G–J).

**FIGURE 7:**
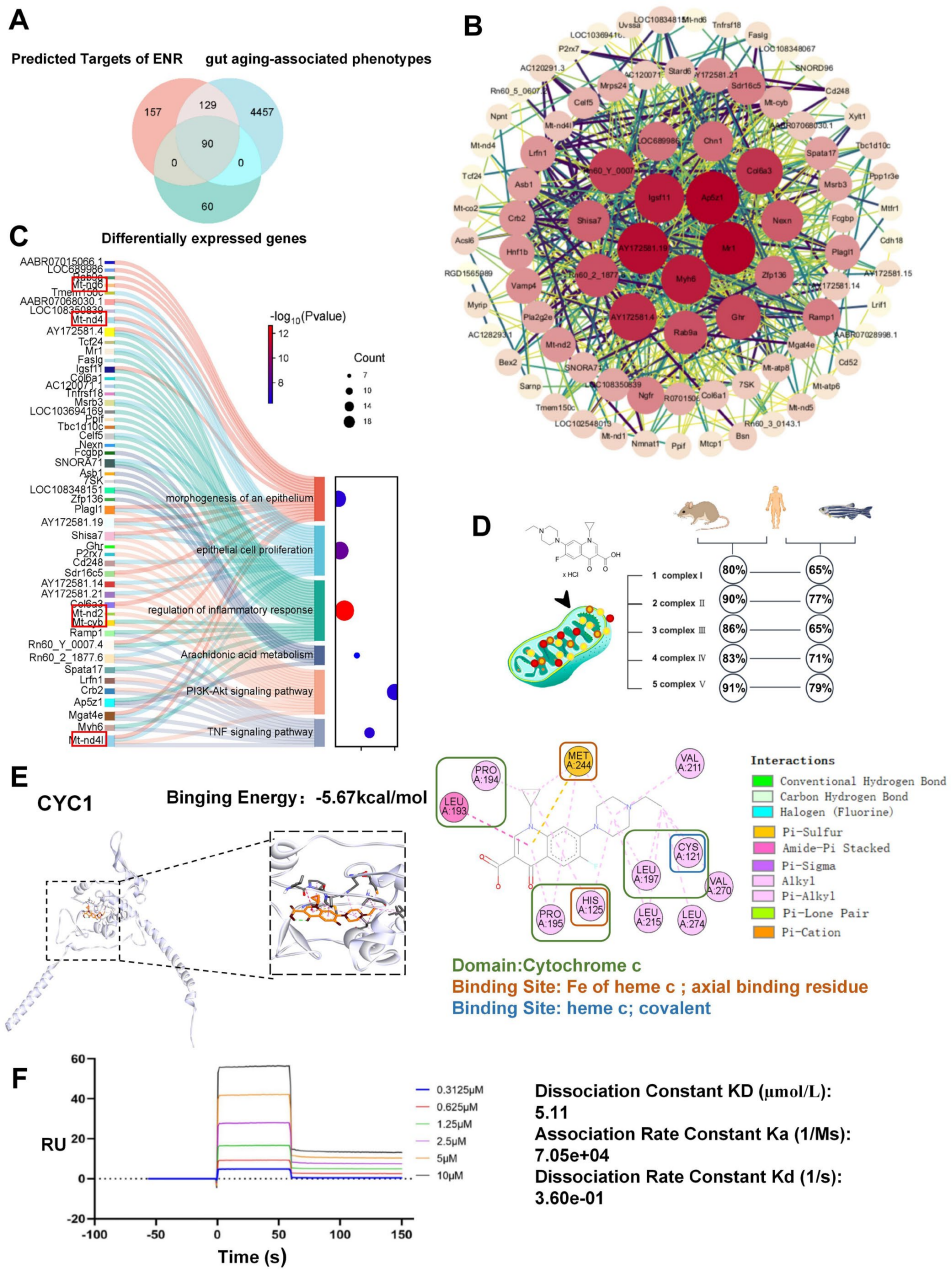
Prediction and validation of ENR targets. (A) Venn diagram illustrating the overlap among predicted ENR targets, inflammatory bowel disease‐related genes, and differentially expressed genes. (B) Protein–protein interaction network representing 90 key target proteins. (C) Sankey diagram displaying enriched KEGG and GO pathways for differentially expressed genes. (D) Comparison of mitochondrial complex protein homology across rats, humans, and zebrafish. (E) Molecular docking simulation diagram of CYC1 protein and ENR. (F) Surface plasmon resonance (SPR) analysis demonstrating binding affinity between ENR and the CYC1 protein.

To further elucidate potential molecular targets underlying ENR‐induced intestinal barrier damage, we performed a comprehensive screening of predicted ENR targets using the Swiss Target Prediction, ChEMBL, and PharmMapper databases, identifying 376 targets. Additionally, we collected 4646 known intestinal aging‐associated targets from GeneCards and Online Mendelian Inheritance in Man (OMIM) databases. Integrating these databases with the differentially expressed genes (DEGs) identified in IEC‐6 cells resulted in 90 overlapping targets between ENR and intestinal aging (Figure 7A). Protein–protein interaction (PPI) network analysis of these common targets revealed 11 mitochondrial function‐associated proteins (Mt‐nd4, Mt‐co2, Mt‐nd2, Mt‐nd1, Mtcp1, Mt‐nd5, Mt‐atp8, Mt‐atp6, Mtfr1, Mt‐cyb, and Mt‐nd6) (Figure 7B). GO and KEGG enrichment analyses demonstrated that these 90 overlapping genes were significantly involved in metabolic pathways, epithelial cell proliferation, morphogenesis, and inflammation‐related pathways, further linking mitochondrial dysfunction to intestinal barrier damage and inflammation (Figure 7C).

To clarify the molecular targets of ENR that influence mitochondrial function, we performed molecular docking simulations focusing on mitochondrial complex subunit proteins and topoisomerases. To ensure translational relevance, we evaluated the protein sequence similarity among humans, rats, and zebrafish. All analyzed proteins exhibited high sequence conservation (over 60%), suggesting comparable functionality across species (Figures S6A and S7A) (Sun et al. 2024). Docking simulations demonstrated potential binding of ENR to active or functional sites of TOP1MT, TOP2B, TOP3A, NDUFV1, and CYC1 proteins (Figures 7E, S6B–D, and S7B,C), implying ENR directly targets mitochondrial complexes and topoisomerases. To experimentally validate these findings, we selected CYC1, the protein showing the most stable binding energy, and measured binding affinity between ENR and CYC1 using SPR (Figures S6B–D and S7B,C). The SPR assay revealed low‐level binding affinity (KD = 5.11 μmol/L) (Figure 7F) (Hu et al. 2024), supporting the potential direct interaction between ENR and mitochondrial proteins. It is important to emphasize that the SPR analysis served to experimentally confirm the binding potential between ENR and CYC1 in a mechanistic context, and was not intended to simulate in vivo exposure concentrations. Collectively, these results support the hypothesis that ENR may directly impair mitochondrial function by interacting with mitochondrial protein subunits such as CYC1, providing a plausible explanation for the observed mitochondrial dysfunction and the accompanying transcriptional changes in mitochondrial‐related genes.

### 3.6 PQQ Supplementation Restores Mitochondrial Function and Reverses ENR‐Induced Gut Damage

**FIGURE 8:**
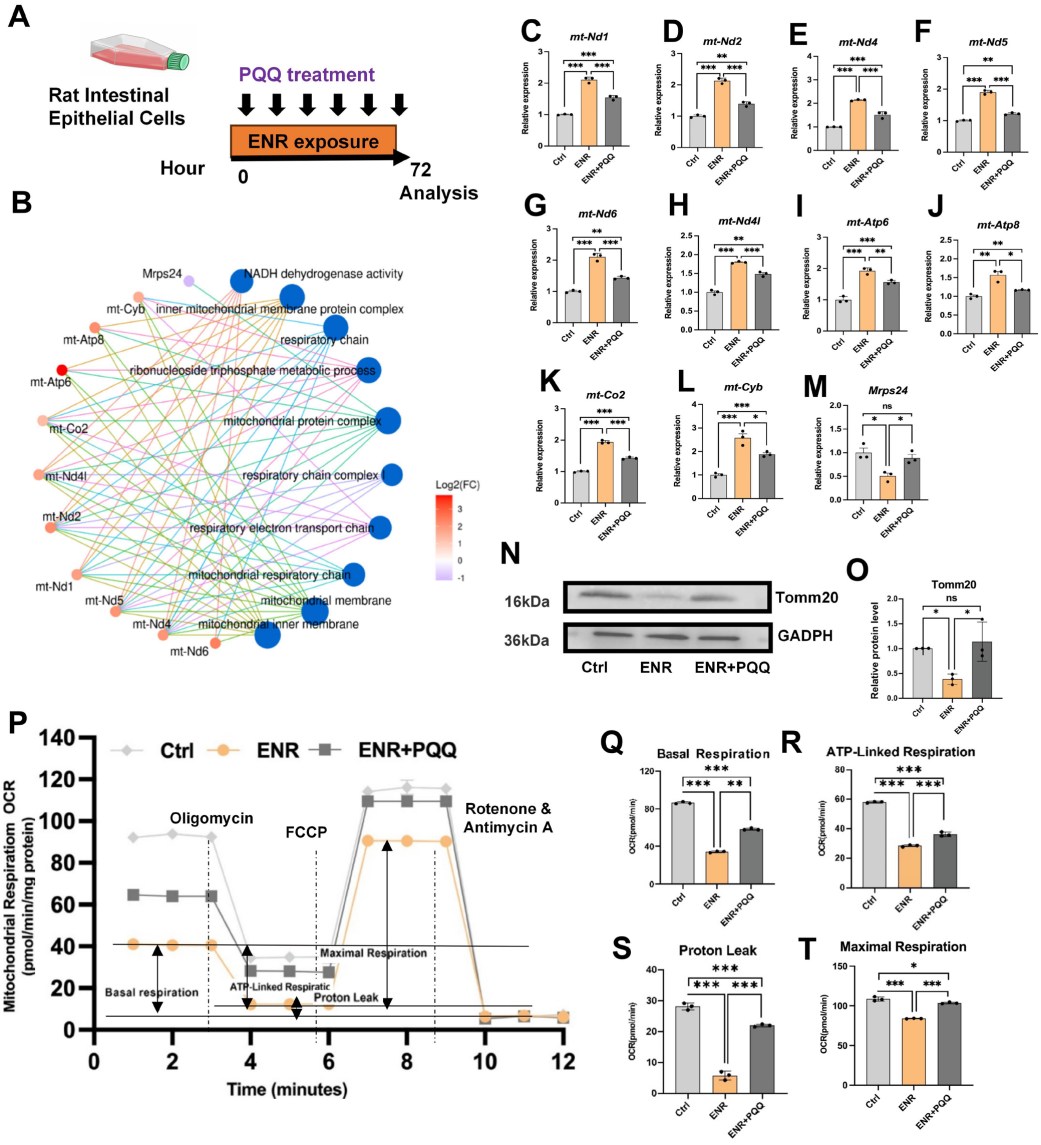
Effects of PQQ treatment on mitochondrial damage induced by ENR exposure in IEC‐6 cells. (A) Experimental design diagram. (B) Network diagram depicting mitochondrial‐related genes and associated biological pathways. (C–M) Differentially expressed genes related to mitochondrial function. (N–O) Protein levels of Tomm20 measured by Western blot analysis. (P) OCR was recorded at baseline and after the sequential injection of oligomycin, FCCP, and a mixture of rotenone and antimycin A. (Q–T) Quantitative analysis of mitochondrial respiration parameters: Basal respiration (Q), ATP‐linked respiration (R), proton leak (S), and maximal respiration (T). Statistical significance among the Control, ENR, and ENR + PQQ groups was assessed using one‐way ANOVA followed by Tukey's multiple‐comparisons test. *p < 0.05, **p < 0.01, ***p < 0.001.

To address mitochondrial dysfunction caused by ENR, we treated IEC‐6 cells simultaneously with ENR and pyrroloquinoline quinone (PQQ), a known mitochondrial function enhancer (Ogawa et al. 2024) (Figure 8A). qRT‐PCR analysis confirmed that PQQ treatment significantly reversed ENR‐induced alterations in mitochondrial function‐related gene expression identified through transcriptomics analysis (Figure 8B–M). Additionally, Western blot results demonstrated that PQQ treatment reversed the ENR‐induced reduction of TOMM20, a mitochondrial outer membrane protein (Figure 8N–O), and improved mitochondrial respiration parameters in IEC‐6 cells (Figure 8P–T).

**FIGURE 9:**
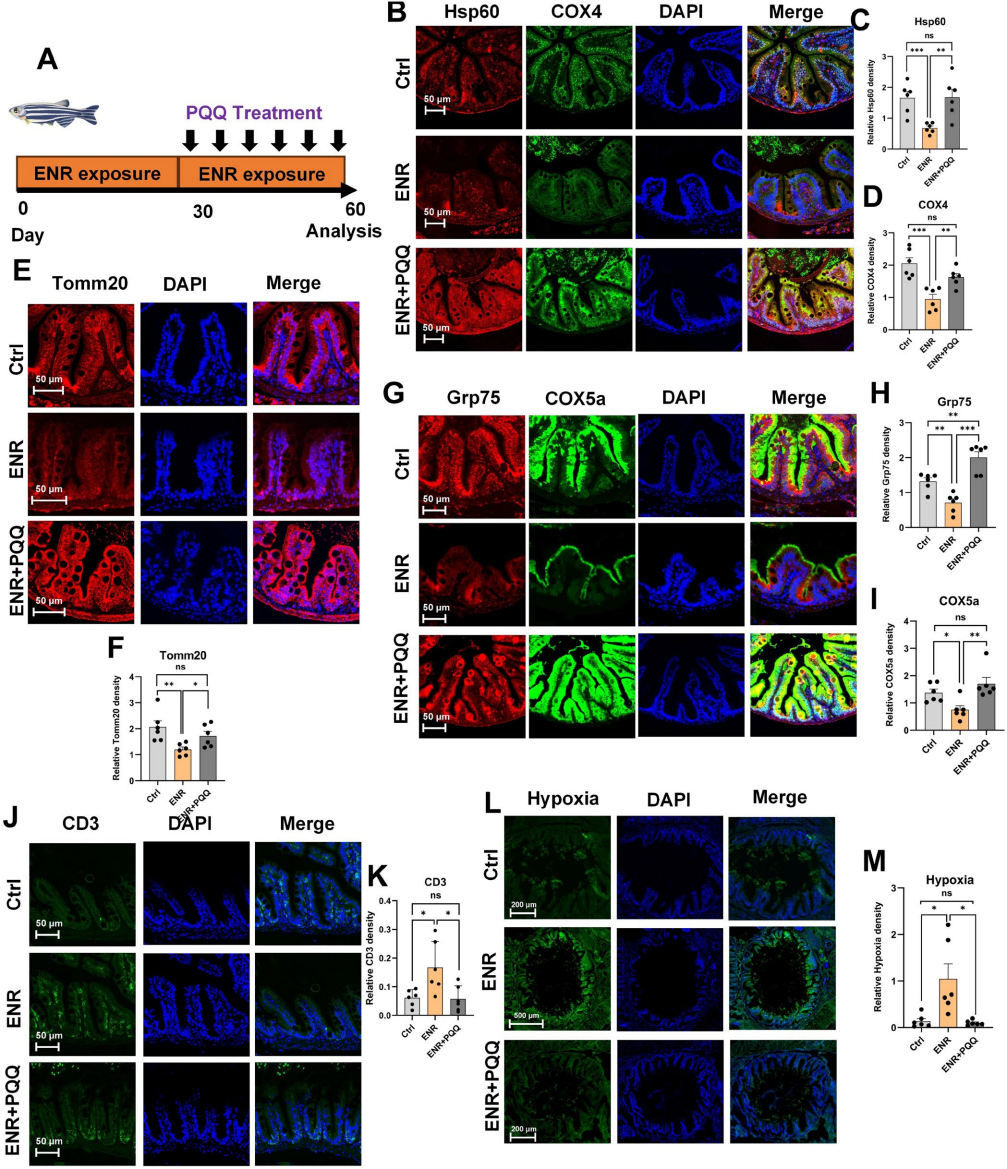
Effects of PQQ treatment on mitochondrial function, inflammation, and hypoxia levels in the intestine following ENR exposure. Experimental design diagram (A). Representative immunofluorescence images of mitochondrial function‐related proteins (Hsp60, Cox4, Tomm20, Grp75, Cox5a) and quantification (B–I). Representative immunofluorescence images of CD3 and quantification (J–K). Representative immunofluorescence images of hypoxia markers and quantification (L, M). Data are presented as the mean ± standard error of the mean. Statistical significance among the Control, ENR, and ENR + PQQ groups was assessed using one‐way ANOVA followed by Tukey's multiple‐comparisons test. *p < 0.05, **p < 0.01, ***p < 0.001.

To further confirm whether targeted mitochondrial restoration could reverse ENR‐induced intestinal damage, we supplemented zebrafish continuously exposed to ENR with PQQ (Figure 9A). Immunofluorescence staining showed that PQQ treatment successfully restored ENR‐induced declines in mitochondrial markers (Tomm20, Hsp60, Cox4, Grp75, Cox5a) within intestinal epithelial cells (Figure 9B–I). Consistently, PQQ also decreased the ENR‐induced accumulation of lymphocytes in intestinal tissues (Figure 9J–K) and reduced intestinal epithelial hypoxia (Figure 9L–M).

**FIGURE 10:**
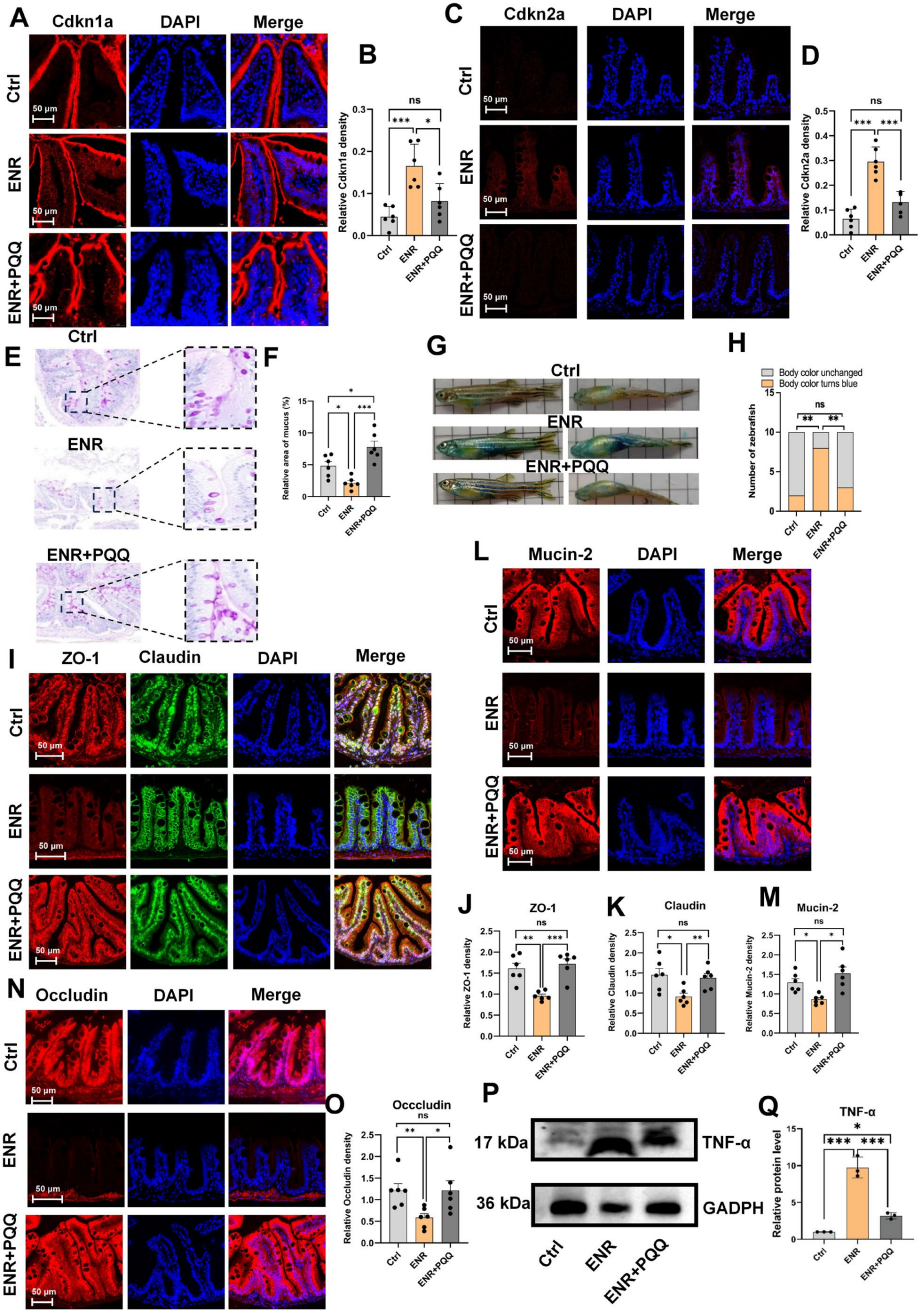
Effects of PQQ treatment on ENR‐induced intestinal barrier damage. (A, B) Representative immunofluorescence images of Cdkn1a and quantification. (C, D) Representative immunofluorescence images of Cdkn2a and quantification. (E, F) Periodic Acid–Schiff (PAS) staining of zebrafish intestinal tissues and quantitative analysis. (G, H) Zebrafish intestinal barrier integrity assessed using the Smurf assay. (I–O) Representative immunofluorescence images of intestinal tight junction proteins (Mucin‐2, Occludin, ZO‐1, Claudin) and quantification. (P, Q) Western blot analysis and quantification of intestinal TNF‐α protein levels. Data are presented as the mean ± standard error of the mean. Statistical significance among the Control, ENR, and ENR + PQQ groups was assessed using one‐way ANOVA followed by Tukey's multiple‐comparisons test. *p < 0.05, **p < 0.01, ***p < 0.001.

At the tissue level, following PQQ treatment, the expression levels of both Cdkn1a and Cdkn2a in the zebrafish intestine were significantly reduced, indicating a partial reversal of the intestinal aging phenotype (Figure 10A–D). The Smurf assay revealed that PQQ supplementation significantly reversed ENR‐induced increases in intestinal permeability (Figure 10G,H). Additionally, PAS staining indicated that PQQ treatment restored the integrity of the intestinal mucus layer damaged by ENR exposure (Figure 10E,F). At the molecular level, PQQ treatment reversed ENR‐induced reductions in intestinal tight junction proteins, including Mucin‐2, Occludin, Zo‐1, and Claudin (Figure 10I–O), and significantly reduced levels of the inflammatory cytokine TNF‐α (Figure 10P,Q).

**FIGURE 11:**
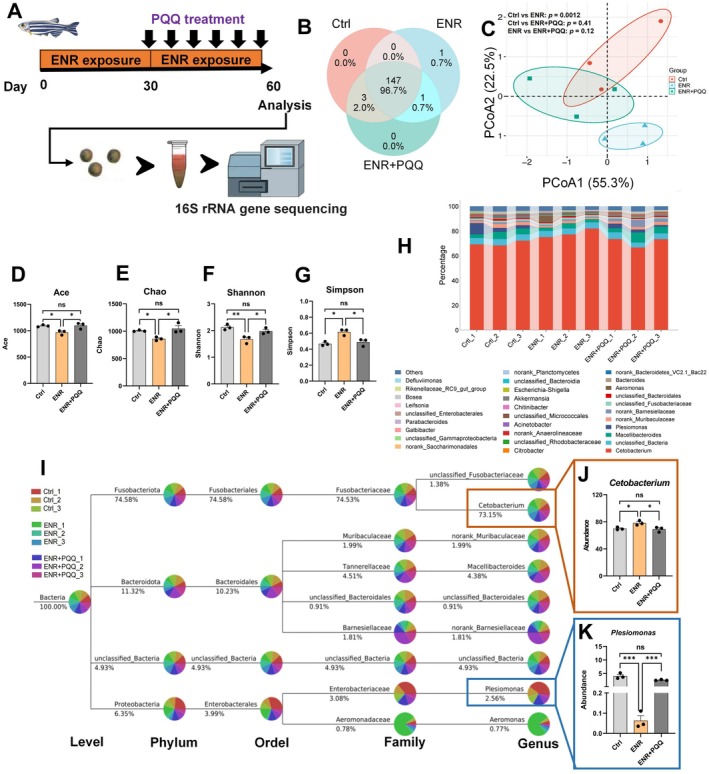
Effects of PQQ treatment on ENR‐induced gut microbiota dysbiosis. (A) Experimental design diagram. (B) Venn diagram showing the overlap of gut microbial genera among Control, ENR, and ENR + PQQ groups. (C) PCA score plot of gut microbiota at genus level. (D–G) Alpha‐diversity index (Ace, Chao, Shannon, Simpson) of gut microbiota at 60 days. (H) Relative abundance of gut microbiota at the genus level across groups. (I) Taxonomic classification across phylum, order, family, and genus levels highlighting *Cetobacterium* and *Plesiomonas*. The percentage shown next to each taxon indicates its overall relative abundance in the corresponding dataset. Each taxonomic node is displayed as a pie chart, and the colored sectors represent the relative contribution of the displayed samples to that taxon. (J–K) Changes in the relative abundance of Cetobacterium (J) and Plesiomonas (K) following ENR exposure and subsequent PQQ treatment. Data are presented as the mean ± standard error of the mean. Statistical significance among the Control, ENR, and ENR + PQQ groups was assessed using one‐way ANOVA followed by Tukey's multiple‐comparisons test. *p < 0.05, **p < 0.01, ***p < 0.001.

To explore the relationship between mitochondrial function recovery and the intestinal microbiome, fecal samples collected from zebrafish after 30 days of PQQ treatment underwent 16S rRNA gene sequencing (Figure 11A). Venn diagram analysis showed greater similarity in bacterial composition between the ENR + PQQ and control groups compared to ENR alone (Figure 11B). Principal coordinates analysis (PCoA) revealed distinct separation between ENR‐exposed and control groups, while the ENR + PQQ group closely overlapped with controls, explaining 88% of the variance (Figure 11C). Furthermore, prolonged ENR exposure (60 days) significantly reduced α‐diversity, highlighting time‐dependent effects of ENR, which were effectively reversed by PQQ supplementation (Figure 11D–G). We showed the top 30 bacterial genera ranked by relative abundance (Figure 11H) and found that PQQ treatment restored the relative abundance of both obligate anaerobes (*Cetobacterium*) and facultative anaerobes (*Plesiomonas*), potentially due to improvement in intestinal hypoxia (Figure 11I). Furthermore, immunofluorescence analysis demonstrated that PQQ supplementation effectively reversed the ENR‐induced elevation of zebrafish intestinal Cdkn1a and Cdkn2a protein levels, highlighting that mitochondrial restoration by PQQ may attenuate the intestinal aging phenotype associated with ENR exposure.

### 3.7 Modulation of Gut Microbiota and Oxidative Status Alleviates Antibiotic‐Induced Physiological Aging and Risk of Diarrhea

**FIGURE 12:**
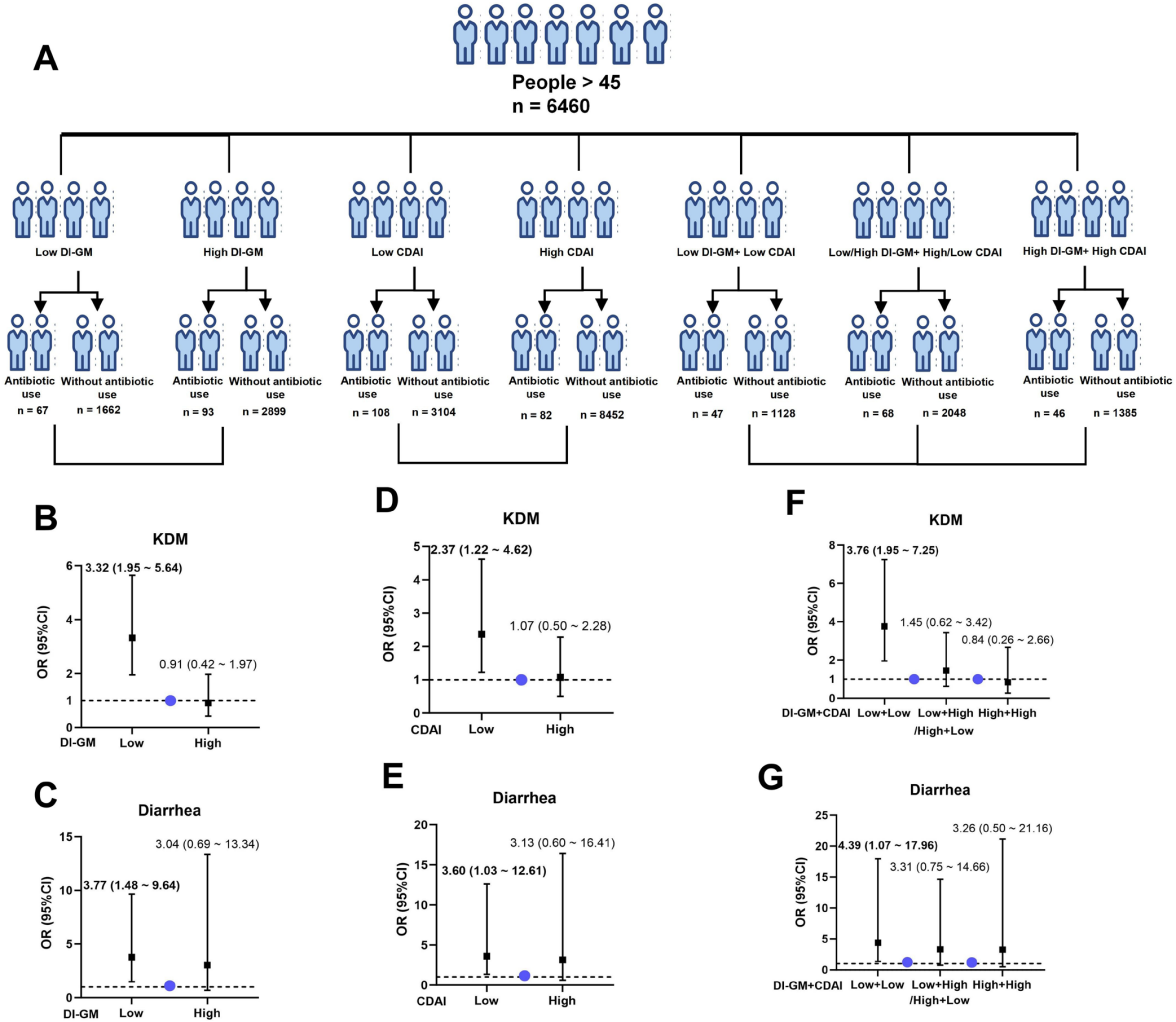
Modulating role of DI‐GM and CDAI in attenuating the associations of antibiotic use with KDM acceleration and diarrhea. (A) Flow chart of participants aged > 45 years stratified by DI‐GM or CDAI (median split) and by recent antibiotic use. (B, C) Associations of recent antibiotic use with KDM acceleration (B) and diarrhea (C) across DI‐GM strata. (D, E) Associations of recent antibiotic use with KDM acceleration (D) and diarrhea (E) across CDAI strata. (F, G) Joint stratification by DI‐GM and CDAI showing the associations of recent antibiotic use with KDM age acceleration (F) and diarrhea (G). Logistic regression models were used, with nonusers serving as the reference group. CDAI, composite dietary antioxidant index; CI, confidence intervals; DI‐GM, dietary index for gut microbiota; KDM, Klemera–Doubal biological age; OR, odds ratio.

Based on the zebrafish experiments above, we found that antibiotics may induce intestinal aging by affecting the gut microbiota and mitochondrial function of intestinal cells. Therefore, we sought to examine whether improving gut microbiota and enhancing antioxidant capacity could mitigate the impact of antibiotics on biological aging and gut‐related health outcomes in human populations. To assess whether improving gut microbiota can reduce antibiotic‐related adverse effects, we stratified the population into low and high dietary index for gut microbiota (DI‐GM) groups (Figure 12A). In the low DI‐GM group, recent antibiotic use was significantly associated with higher odds of KDM acceleration (OR = 3.32, 95% CI: 1.95–5.64) and self‐reported diarrhea (OR = 3.77, 95% CI: 1.48–9.64), whereas no significant associations were observed in the high DI‐GM group (Figure 12B,C). These findings suggest that a healthier microbiota may offer protection against antibiotic‐induced damage.

To evaluate whether elevated antioxidant defenses can attenuate the detrimental effects associated with antibiotic exposure, we further analyzed oxidative stress by dividing participants into low and high composite dietary antioxidant index (CDAI) groups. In the low CDAI group, recent antibiotic use was significantly associated with higher odds of KDM acceleration (OR = 2.37, 95% CI: 1.22–4.62) and self‐reported diarrhea (OR = 3.60, 95% CI: 1.03–12.61), while no significant associations were found in the high CDAI group (Figure 12D,E), indicating that higher antioxidant status may also confer resilience.

Considering the combined impact of microbiota and oxidative stress, we classified individuals into double‐low, single‐low, and double‐high groups. Only the double‐low group showed significant associations of recent antibiotic use with higher odds of KDM acceleration (OR = 3.76, 95% CI: 1.95–7.25) and self‐reported diarrhea (OR = 4.39, 95% CI: 1.07–17.96), whereas no significant associations were observed in the other groups (Figure 12F,G).

Together, these results indicate that both gut microbiota and oxidative balance independently reduce antibiotic‐associated risks, and their combined optimization provides the greatest protective effect against antibiotic‐induced physiological aging and gastrointestinal symptoms.

## 4 Discussion

Despite the well‐documented impact of antibiotics on gut microbiota and host metabolism, their long‐term effects as environmental factors on aging remain incompletely characterized (Hanna et al. 2023; Argentieri et al. 2025). Specifically, whether chronic environmental antibiotic exposure accelerates biological aging through combined mechanisms such as mitochondrial dysfunction, microbial dysbiosis, and inflammation has not been systematically resolved (Miller and Singer 2022; Li et al. 2023). Our research indicates that chronic exposure to environmental levels of ENR may contribute to intestinal aging by disrupting epithelial barrier integrity, altering gut microbial composition, and impairing mitochondrial function (Urbauer et al. 2024; Neurath et al. 2025). Mechanistically, ENR‐induced injury appears consistent with a mitochondria–microbiota axis in which epithelial mitochondrial dysfunction compromises barrier and oxygen homeostasis, reshaping the microbial niche and promoting inflammation (Rath and Haller 2022; Savage et al. 2024). Notably, restoration of mitochondrial function alleviated ENR‐induced intestinal damage and reversed aging‐associated phenotypes, highlighting mitochondrial protection as a plausible therapeutic avenue (Rath and Haller 2022; Yan et al. 2024). Together, these findings support the concept that environmental antibiotic exposure may serve as a modifiable risk factor for intestinal aging and underscore the importance of maintaining mitochondrial and microbial homeostasis in gut health (Salazar et al. 2023; Argentieri et al. 2025).

Human exposure to antibiotics via routine medical care and residues in the food supply may represent an important extrinsic driver of organismal aging and intestinal dysfunction (Ben et al. 2022; Argentieri et al. 2025). Our population‐based analysis showed that antibiotic use was associated with higher biological aging levels and an increased risk of diarrhea, consistent with broader evidence linking antibiotic exposure to adverse gut outcomes (Goodman et al. 2021; Gao et al. 2023). These associations remained significant after adjustment for infection, supporting a potential direct contribution of antibiotics to aging‐related processes and intestinal health (Gao et al. 2023; Li et al. 2023). NHANES defines antibiotic exposure based on self‐reported prescription antibiotic use within the previous 30 days and therefore primarily captures recent, short‐course therapeutic antibiotic use rather than quantified cumulative exposure to chronic low‐dose environmental residues, or to ENR specifically (Frenk et al. 2016). This distinction is important because short‐term therapeutic antibiotic use is typically indication‐driven and administered over a limited period at pharmacologically active doses, whereas environmental antibiotic exposure is generally inadvertent, occurs at lower doses, and tends to be more continuous or recurrent over time through background residues in food and drinking water (Ben et al. 2022; Hu et al. 2022; Wang et al. 2022). Nevertheless, the importance of the NHANES analysis lies in showing that antibiotic exposure is associated with aging‐related and gut‐related phenotypes in a real‐world human population, thereby providing translational relevance and a human‐centered rationale for our subsequent mechanistic experiments. In the subsequent experiments, our zebrafish and cellular models directly tested sustained, environmentally relevant ENR exposure under controlled conditions and supported a mitochondria–hypoxia–barrier pathway in which microbiota remodeling primarily amplifies inflammation during chronic low‐dose exposure.

At the mechanistic level, antibiotics can disrupt the gut microbiota and its metabolite profile, thereby promoting mucosal immune activation and intestinal inflammation, which may contribute to diarrhea risk (Duan et al. 2022; Neurath et al. 2025). However, our FMT experiments indicate that ENR‐altered microbiota is sufficient to transmit intestinal inflammatory features but does not recapitulate barrier disruption or mucosal hypoxia in recipients (Figure 5). Notably, compromised barrier integrity in our dataset is more tightly linked to epithelial mitochondrial dysfunction and hypoxia, and mitochondrial restoration by PQQ concurrently rescues permeability, tight‐junction, and mucus phenotypes (Figures 6, 7, 8, 9, 10, 11). Together, these results support a model in which microbiota dysbiosis primarily amplifies inflammation via metabolite shifts, whereas epithelial mitochondrial injury represents a proximal driver of hypoxia‐associated barrier breakdown during chronic ENR exposure. Consistently, antibiotic‐associated mitochondrial dysfunction can amplify cellular stress responses and senescence programs (including Cdkn1a and Cdkn2a), thereby reinforcing inflammatory and barrier‐impairment processes (Miller and Singer 2022; Rath and Haller 2022). Our findings further support the notion that environmental antibiotic exposure may contribute to this aging‐like intestinal phenotype (Bhatt and Chatterjee 2022; Hanna et al. 2023).

In contrast to therapeutic dosing, environmental low‐dose antibiotic exposure may impose sustained, low‐grade perturbations at the microbiota–host interface over extended time windows (Bhatt and Chatterjee 2022; Hanna et al. 2023). This temporal feature is also reflected in our data: we observed no appreciable change in microbial α‐diversity at 30 days, but a marked decline by 60 days, suggesting that chronic low‐dose ENR exposure reshapes the gut microbiota in a progressive manner. The preservation of mucosal barrier integrity in the ENR‐FMT group further suggests that transplanted microbiota may influence host phenotypes primarily through metabolite‐mediated inflammatory signaling rather than by directly initiating barrier failure (Bosco and Noti 2021; Bradley and Haran 2024). This interpretation is consistent with the possibility that early compositional and functional shifts precede a later collapse in overall diversity under prolonged exposure. In support of this view, we identified alterations in amino acid and lipid metabolism that are frequently linked to aging and age‐related microbiome remodeling (Salazar et al. 2023; Bradley and Haran 2024). Gut microbial metabolites have been implicated in the regulation of host cellular stress and senescence programs (Bradley and Haran 2024; Yang et al. 2025), and altered amino acid availability can further shape epithelial stress responses and inflammatory tone in the gut (Li et al. 2023; Neurath et al. 2025). Nutritional supplementation strategies, including specific amino acids, have been reported to ameliorate epithelial injury and inflammation in relevant contexts, supporting reduced amino acid availability as one plausible contributor to microbiota‐associated inflammation following ENR exposure (Baier et al. 2020; Ding et al. 2023).

Notably, ENR‐induced intestinal inflammation in our model was accompanied by pronounced gut hypoxia, consistent with the concept that inflammation can perturb epithelial oxygenation and thereby reshape the microbial ecosystem (Savage et al. 2024; Neurath et al. 2025). Altered epithelial oxygen states can shift the balance between obligate and facultative anaerobes and influence colonization resistance and disease trajectories (Savage et al. 2024; Van Hul et al. 2024). Antibiotic exposure is a major risk factor for loss of colonization resistance and for Clostridioides difficile infection, particularly in older adults, with clinical guidance increasingly incorporating microbiota‐based therapeutic strategies (Johnson et al. 2021; Peery et al. 2024). Our findings therefore support a model in which chronic low‐dose ENR exposure induces epithelial and oxygenation changes that drive microbiota remodeling, metabolic dysfunction, and inflammation, accelerating gut aging phenotypes (Salazar et al. 2023; Savage et al. 2024).

Accumulating evidence indicates that mitochondrial dysfunction amplifies inflammatory signaling and cellular stress responses, and these changes are closely linked to disruption of intestinal epithelial homeostasis and barrier integrity (Guerbette et al. 2022; Rath and Haller 2022). Our study identifies mitochondrial dysfunction as a central mechanism of ENR‐induced intestinal barrier damage, supported by the observation that mitochondrial perturbation in the intestinal epithelium can drive microbiota‐dependent injury programs (Rath and Haller 2022; Urbauer et al. 2024). Molecular docking and SPR analyses in our work suggest that ENR can bind mitochondrial respiratory chain subunits, providing a plausible direct route to mitochondrial impairment. Such mitochondrial perturbation is widely associated with increased ROS, suppressed ATP production, and stress pathway activation, which can amplify inflammatory responses and barrier dysfunction (Miller and Singer 2022; Rath and Haller 2022). Despite growing evidence linking mitochondrial dysfunction to aging and gut pathophysiology, targeted strategies that translate this biology into barrier‐protective interventions remain comparatively underdeveloped (Guerbette et al. 2022; Rath and Haller 2022). Pyrroloquinoline quinone (PQQ) has been reviewed as a bioactive compound with potential mitochondrial‐protective and redox‐modulating effects, and recent work supports its ability to engage antioxidant/anti‐senescence pathways (e.g., Nrf2‐related programs) in aging contexts (Xue et al. 2024; Yan et al. 2024). In our study, PQQ supplementation restored mitochondrial function and reversed ENR‐induced intestinal injury, supporting mitochondria‐targeted mitigation as a promising direction (Rath and Haller 2022; Yan et al. 2024).

Our population‐based analyses further corroborated an antibiotic–gut microbiota–mitochondria/oxidative stress–aging pathway suggested by our experimental work. Clinically, probiotics and, in selected indications, fecal microbiota–based therapies have been evaluated to reduce antibiotic‐associated dysbiosis and diarrhea risk, and recent syntheses and guidelines provide updated evidence frameworks for such approaches (Mullish et al. 2024). Our data further indicate that improving gut microbiota resilience and antioxidant capacity may serve as complementary strategies to buffer the impact of chronic, low‐dose environmental antibiotic exposure and thereby attenuate intestinal aging processes (Salazar et al. 2023; Argentieri et al. 2025).

In conclusion, our work identifies chronic exposure to environmentally relevant levels of enrofloxacin as a plausible and modifiable driver of intestinal aging. By combining population‐based analyses with zebrafish and cellular models, we delineate an antibiotic–mitochondria–microbiota axis through which low‐dose antibiotic residues can gradually undermine gut homeostasis and contribute to aging‐related intestinal dysfunction (Rath and Haller 2022; Salazar et al. 2023). These findings extend the concept of inflammaging to include pharmaceutical pollution, highlighting antibiotics as not only antimicrobial agents but also potential accelerators of biological aging in the gut (Hanna et al. 2023; Li et al. 2023). Importantly, our data suggest that preserving mitochondrial integrity, enhancing gut microbiota resilience, and improving dietary antioxidant status may help buffer the long‐term impact of environmental antibiotic exposure (Bradley and Haran 2024; Yan et al. 2024). This framework provides a mechanistic basis for incorporating antibiotic pollution into aging risk assessment and for developing targeted prevention and intervention strategies (Hanna et al. 2023; Argentieri et al. 2025).

## 5 Limitations

This study has several limitations. First, although zebrafish provide a convenient vertebrate model to interrogate gut barrier integrity, microbiota changes, and mitochondrial function, interspecies differences in intestinal physiology, immune organization, and aging trajectories limit direct extrapolation to humans. Our ENR exposure paradigm (single compound, fixed dose, and duration) also simplifies the complex, mixed, and lifelong environmental antibiotic exposures encountered in real‐world settings, and the FMT and PQQ regimens were optimized for experimental rather than clinical use. Second, the NHANES analysis is cross‐sectional, precluding causal inference, and relies on 30‐day self‐reported antibiotic use and diarrhea, while KDM age acceleration, DI‐GM, and CDAI are surrogate indices rather than direct measures of microbiota composition, antioxidant capacity, or cumulative exposure. Future research should integrate longitudinal human cohorts with deep multi‐omics profiling and mechanistic studies in mammalian models, including interventional trials targeting mitochondria and microbiota, to substantiate and extend the pathways proposed here.

## Author Contributions

Kan Yu contributed to the conceptualization, data curation, formal analysis, methodology, and writing – original draft. Nengzheng Wang contributed to the data curation, methodology, and writing – review and editing. Xinyi Huang contributed to the investigation. Yushu Qiu contributed to the data curation. Xuejian He and Xiaofeng Zhu assisted in the project administration. Xiaolan Zhou assisted in the project administration and Visualization. Peng Yu assisted in the project administration. Gang Wei assisted in the project administration. Yan Pi and Ting Ni contributed to the conceptualization, funding acquisition, supervision, and writing – review and editing.

## Funding

This work was supported by National Key Research and Development Program of China, 2023YFC3603300; Shanghai Municipal Science and Technology Major Project, 2023SHZDZX02.

## Conflicts of Interest

The authors declare no conflicts of interest.

## Supporting information


**Figure S1:** (A) Schematic diagram of the IEC‐6 cell experiment. (B, C) Relative transcription levels of the inflammatory markers *il‐6* and *tnf‐α* in IEC‐6 cells after ENR exposure. (D) Schematic diagram of the zebrafish ENR exposure experiment. (E–J) Relative transcription levels of inflammatory factors in zebrafish intestinal tissue after ENR exposure. (K–R) qRT‐PCR validation of intestinal barrier‐ and hypoxia‐related genes in zebrafish intestinal tissue after ENR exposure, including *muc2.2* (K), *tjp1a* (L), *tjp1b* (M), *cldn1* (N), *oclna* (O), *oclnb* (P), *hif1aa* (Q), and *hif1ab* (R). Data are presented as mean ± SEM (*n* = 3 biological replicates per group). Statistical significance was assessed using two‐tailed Student's *t*‐test. *p < 0.05, **p < 0.01, ***p < 0.001.
**Figure S2:** (A–D) Alpha‐diversity index (Ace, Chao, Shannon, Simpson) of gut microbiota at 30 days. (E) LEfSe analysis of the intestinal microbiome at the genus level following 30 days of ENR exposure. (F) Heatmap depicting genes associated with amino acid metabolism in the intestinal microbiome. (G) Heatmap illustrating genes related to lipid metabolism in the intestinal microbiome.
**Figure S3:** Metagenomic analysis of the gut microbiome following 30 days of ENR exposure. (A) Heatmap showing genes related to energy metabolism in the gut microbiome. (B) Heatmap displaying genes associated with carbohydrate metabolism in the gut microbiome. (C) Correlation analysis between the abundance of metabolic function‐related genes and differential metabolites in the gut.
**Figure S4:** Metabolite analysis of intestinal tissue and its contents. (A) Amino acid and peptide metabolites. (B) Lipid metabolites. (C) Pro‐inflammatory metabolites and (D) anti‐inflammatory metabolites. (E) Sankey plot illustrating the relationship between gut microbiota, metabolic function‐related genes, and differential metabolites (p < 0.05, |β| > 0.9). Data are presented as the mean ± standard error of the mean. Statistical significance was assessed using Student's t‐test. *p < 0.05, **p < 0.01, ***p < 0.001.
**Figure S5:** (A) Flowchart of the fecal microbiota transplantation experiment. (B) Total bacterial load in zebrafish feces was quantified by qRT‐PCR. (C, D) Relative abundances of Cetobacterium (C) and Plesiomonas (D) were measured at day 30 post‐FMT. Abundances were normalized to total bacteria 16S rRNA gene levels in each sample. (E–H) Representative immunofluorescence images showing mitochondrial function‐related proteins (Tomm20, Hsp60) and their quantification. Data are presented as the mean ± standard error of the mean. Statistical significance was assessed using Student's t‐test. *p < 0.05, **p < 0.01, ***p < 0.001.
**Figure S6:** (A) Homology comparison of topoisomerase proteins between rat, human, and zebrafish. (B) Molecular docking simulation of TOP1MT protein and enrofloxacin (ENR). (C) Molecular docking simulation of TOP2B protein and ENR. (D) Molecular docking simulation of TOP3A protein and ENR.
**Figure S7:** (A) Homology comparison of mitochondrial complex proteins between rat, human, and zebrafish. (B) Molecular docking simulation of NDUFV1 protein and enrofloxacin (ENR). (C) Molecular docking simulation of CYC1 protein and ENR.
**Figure S8:** (A) Experimental flow chart for 16S rRNA gene sequencing and analysis. (B) Genus‐level LEfSe analysis comparing the ENR and control groups. (C) Genus‐level LEfSe analysis comparing the ENR + PQQ and control groups.
**Figure S9:** Venn diagram illustrating the overlap among predicted antibiotics targets and Mitochondrial dysfunction‐related genes.
**Figure S10:** Representative Western blot analysis of TOMM20 and TNF‐α protein expression. (A) Whole‐membrane Western blot image showing the expression of TOMM20 in three samples. (B) Whole‐membrane Western blot image of TNF‐α expression.
**Figure S11:** Flowchart of participant inclusion and exclusion in the NHANES analysis.

**Table S1:** Baseline characteristics by antibiotic use.
**Table S2:** Stratified analysis of antibiotic use–related KDM and diarrhea by age group.
**Table S3:** DI‐GM and CDAI treatments in relation to antibiotic‐associated KDM and diarrhea in individuals aged 45 and above.
**Table S4:** Components and scoring criteria of DI‐GM in NHANES.
**Table S5:** Primer sequences used for qRT‐PCR in zebrafish and IEC‐6 cells.

## Data Availability

All relevant datasets generated and/or analyzed during this study are publicly available as follows: Metagenomic sequencing data have been deposited in the Genome Sequence Archive (GSA) under the accession link: https://ngdc.cncb.ac.cn/gsa/s/Gp5w810o. Transcriptomic data are available in the GSA database at: https://ngdc.cncb.ac.cn/gsa/s/4E3fR0ZX. 16S rRNA gene sequencing data are accessible via the GSA portal: https://ngdc.cncb.ac.cn/gsa/s/2YVbi798. Metabolomics data are available in the OMIX database at: https://ngdc.cncb.ac.cn/omix/preview/5tZlfvXt.
