# Correction to “Environmental Enrofloxacin Exposure as a Modifiable Driver of Mitochondria‐Mediated Intestinal Aging and Barrier Dysfunction”

**DOI:** 10.1111/acel.70598

**Published:** 2026-06-25

Yu, K. , N. Wang , X. Huang , et al. (2026). Environmental Enrofloxacin Exposure as a Modifiable Driver of Mitochondria‐Mediated Intestinal Aging and Barrier Dysfunction. Aging Cell 25, no. 5: e70526. 10.1111/acel.70526.42059464 PMC13130356

In the legend of Figure 6, the first sentence, “Impact of ENR exposure on zebrafish intestinal epithelial cells,” was incorrect. This should have read: “Impact of ENR exposure on rat intestinal epithelial cells.” The correct figure is as follows:

**FIGURE 6:**
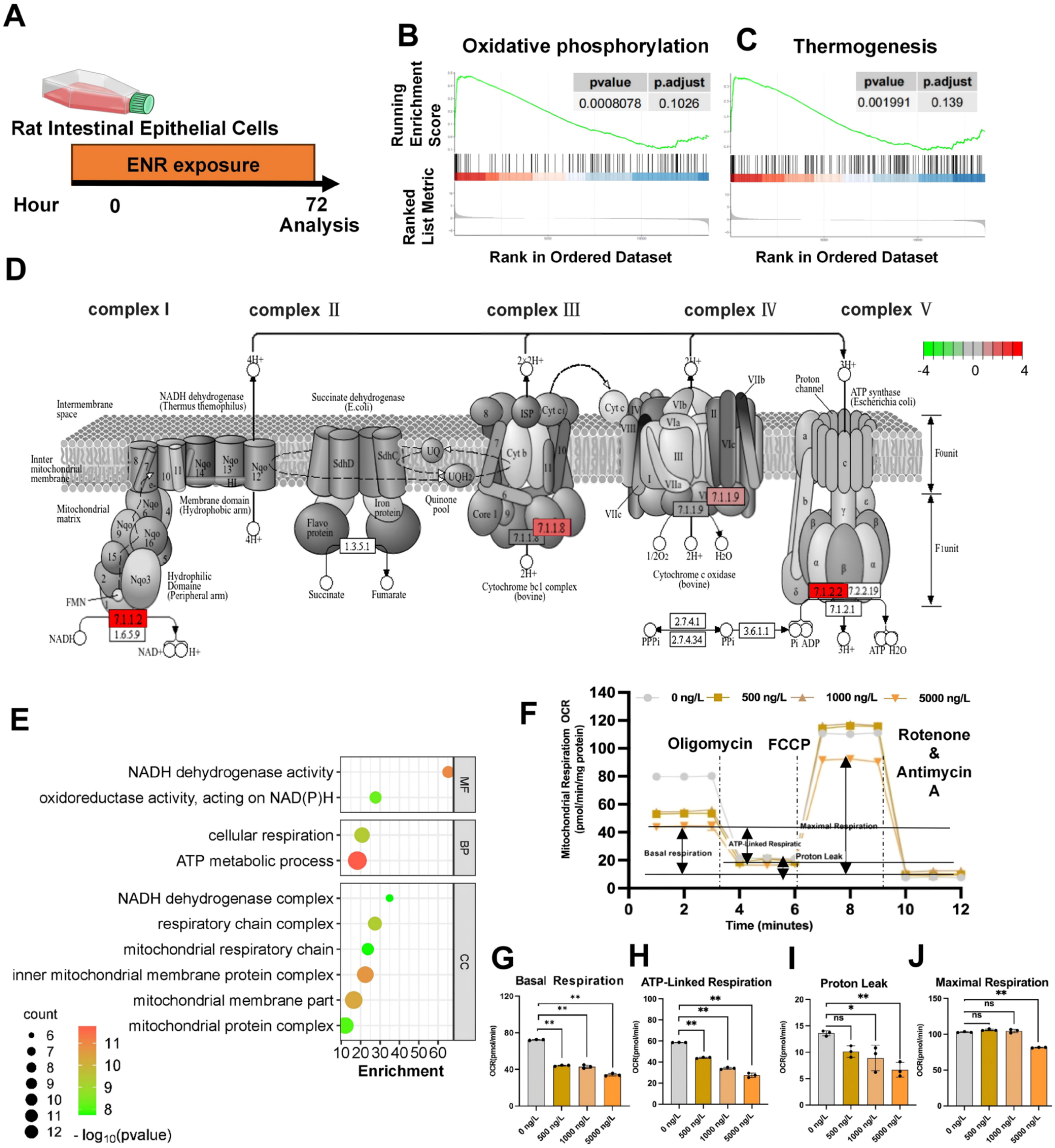
Impact of ENR exposure on rat intestinal epithelial cells. (A) Overview of experimental procedures used to examine intestinal epithelial responses after ENR exposure. (B, C) Gene Set Enrichment Analysis (GSEA) plots highlighting enrichment in oxidative phosphorylation (B) and thermogenesis pathways (C). (D) Products of genes with significant changes in the oxidative phosphorylation pathway. (E) Gene Ontology (GO) enrichment analysis of mitochondrial function‐related pathways significantly impacted by ENR exposure. (F) Mitochondrial oxygen consumption rate (OCR) was recorded at baseline and after the sequential injection of oligomycin, FCCP, and a mixture of rotenone and antimycin A. (G, J) Quantitative analyses of mitochondrial respiratory parameters: Basal respiration (G), ATP‐linked respiration (H), proton leak (I), and maximal respiration (J). Data are presented as the mean ± standard error of the mean. Statistical significance was assessed using one‐way ANOVA. *p < 0.05, **p < 0.01, ***p < 0.001.

In Figure 9J, the merged images for the Ctrl and ENR groups in the Merge column were inadvertently placed in the wrong order during figure preparation. The correct figure is as follows:

**FIGURE 9:**
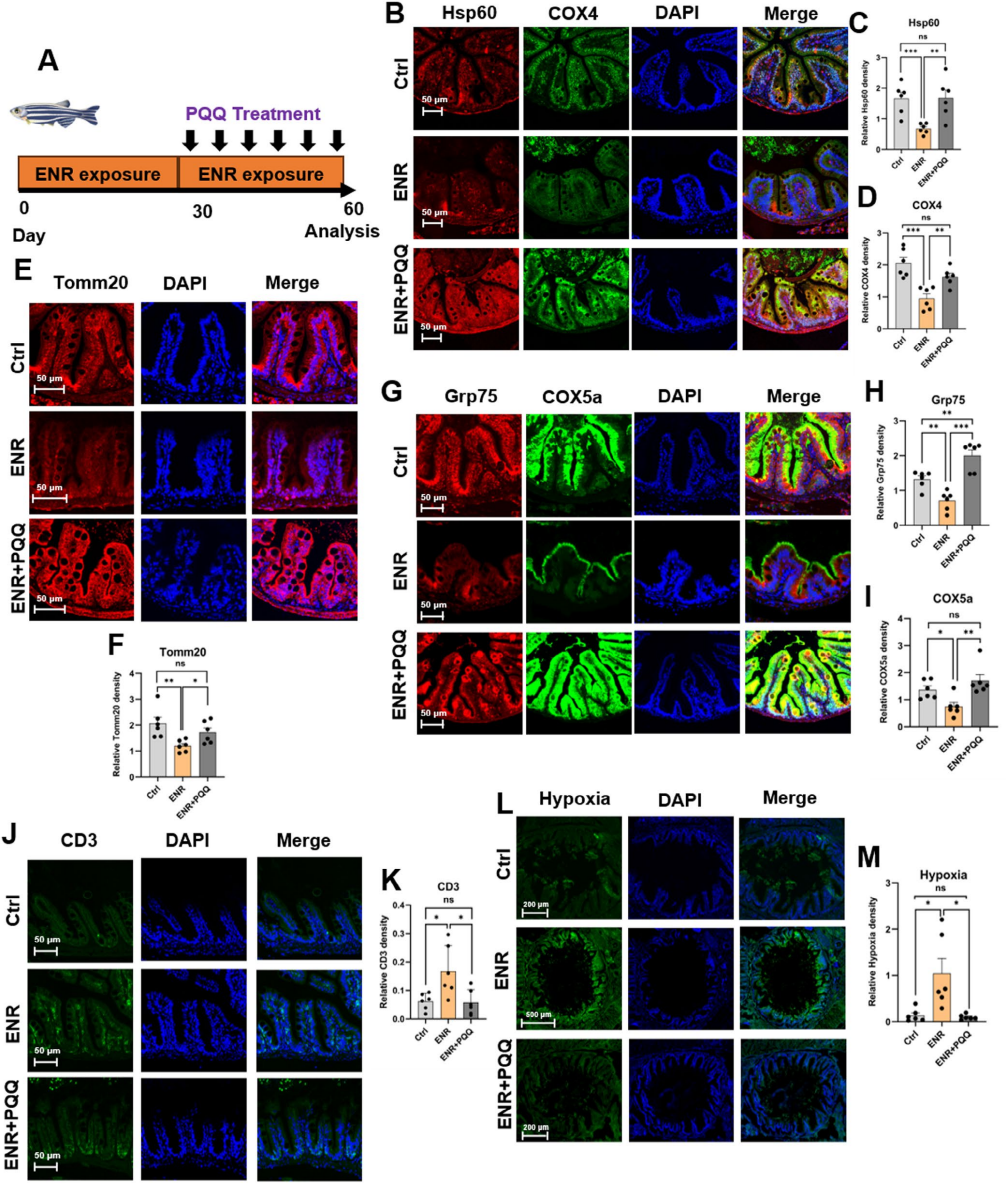
Effects of PQQ treatment on mitochondrial function, inflammation, and hypoxia levels in the intestine following ENR exposure. Experimental design diagram (A). Representative immunofluorescence images of mitochondrial function‐related proteins (Hsp60, Cox4, Tomm20, Grp75, Cox5a) and quantification (B–I). Representative immunofluorescence images of CD3 and quantification (J–K). Representative immunofluorescence images of hypoxia markers and quantification (L, M). Data are presented as the mean ± standard error of the mean. Statistical significance among the Control, ENR, and ENR + PQQ groups was assessed using one‐way ANOVA followed by Tukey's multiple‐comparisons test. *p < 0.05, **p < 0.01, ***p < 0.001.

These errors were introduced during the figure assembly process and do not affect the corresponding single‐channel images, the ENR+PQQ group, the quantitative analysis shown in Figure 9K, or the conclusions of the article.

We apologize for these errors.

