# Supplementary material for: Environmental Enrofloxacin Exposure as a Modifiable Driver of Mitochondria‐Mediated Intestinal Aging and Barrier Dysfunction
Source: Aging Cell. 2026 Apr 30;25(5):e70526. doi: 10.1111/acel.70526 (PMC13130356; doi:10.1111/acel.70526)
Supplement: Supplementary file 1 — Figure S1: (A) Schematic diagram of the IEC‐6 cell experiment. (B, C) Relative transcription levels of the inflammatory markers il‐6 and tnf‐α in IEC‐6 cells after ENR exposure. (D) Schematic diagram of the zebrafish ENR exposure experiment. (E–J) Relative transcription levels of inflammatory factors in zebrafish intestinal tissue after ENR exposure. (K–R) qRT‐PCR validation of intestinal barrier‐ and hypoxia‐related genes in zebrafish intestinal tissue after ENR exposure, including muc2.2 (K), tjp1a (L), tjp1b (M), cldn1 (N), oclna (O), oclnb (P), hif1aa (Q), and hif1ab (R). Data are presented as mean ± SEM (n = 3 biological replicates per group). Statistical significance was assessed using two‐tailed Student's t‐test. *p < 0.05, **p < 0.01, ***p < 0.001. Figure S2: (A–D) Alpha‐diversity index (Ace, Chao, Shannon, Simpson) of gut microbiota at 30 days. (E) LEfSe analysis of the intestinal microbiome at the genus level following 30 days of ENR exposure. (F) Heatmap depicting genes associated with amino acid metabolism in the intestinal microbiome. (G) Heatmap illustrating genes related to lipid metabolism in the intestinal microbiome. Figure S3: Metagenomic analysis of the gut microbiome following 30 days of ENR exposure. (A) Heatmap showing genes related to energy metabolism in the gut microbiome. (B) Heatmap displaying genes associated with carbohydrate metabolism in the gut microbiome. (C) Correlation analysis between the abundance of metabolic function‐related genes and differential metabolites in the gut. Figure S4: Metabolite analysis of intestinal tissue and its contents. (A) Amino acid and peptide metabolites. (B) Lipid metabolites. (C) Pro‐inflammatory metabolites and (D) anti‐inflammatory metabolites. (E) Sankey plot illustrating the relationship between gut microbiota, metabolic function‐related genes, and differential metabolites (p < 0.05, |β| > 0.9). Data are presented as the mean ± standard error of the mean. Statistical significance was assessed usin [file ACEL-25-e70526-s002.docx]

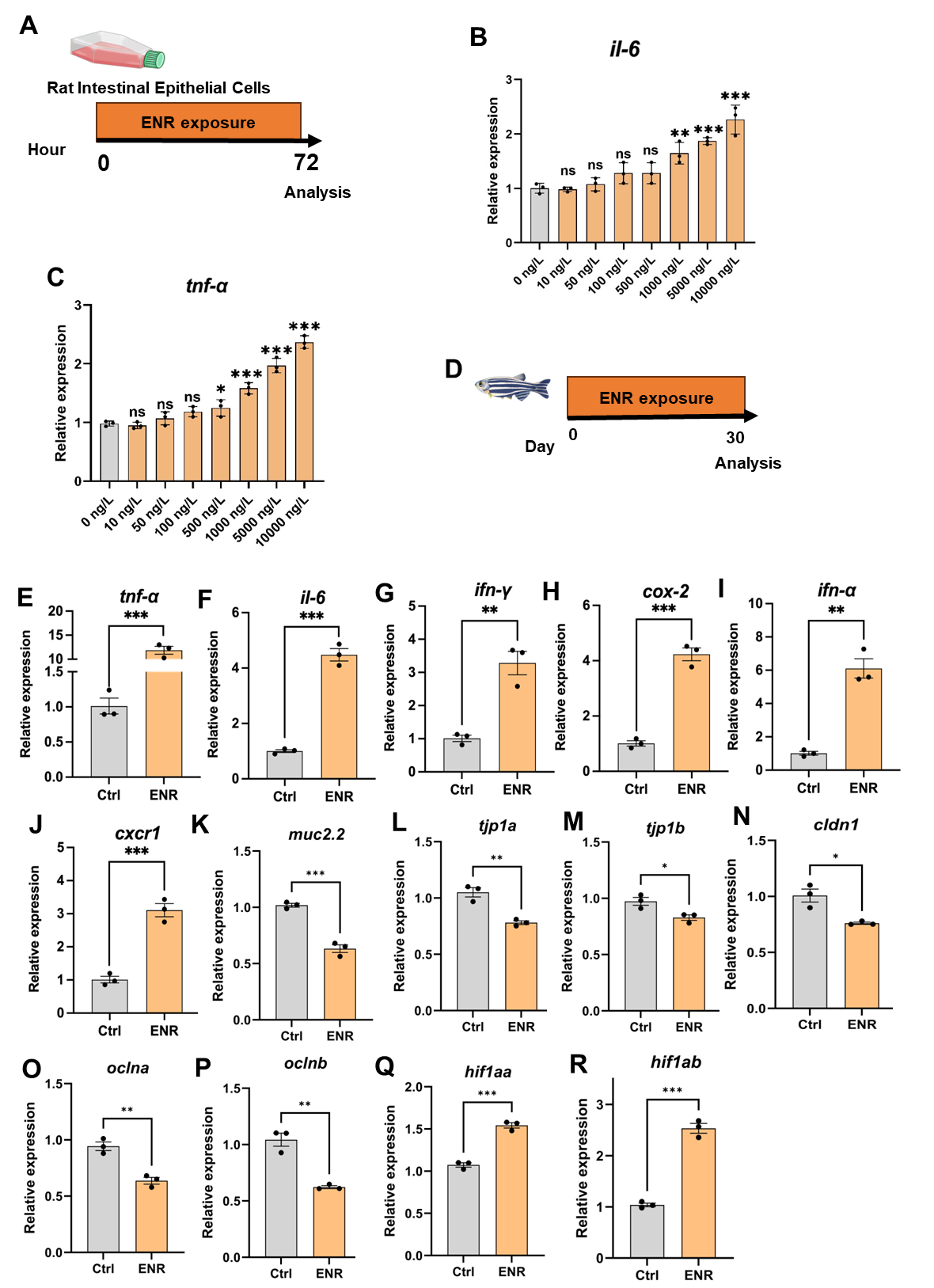


**Supplementary Figure 1.** (A) Schematic diagram of the IEC-6 cell experiment. (B, C) Relative transcription levels of the inflammatory markers *il-6* and *tnf-α* in IEC-6 cells after ENR exposure. (D) Schematic diagram of the zebrafish ENR exposure experiment. (E–J) Relative transcription levels of inflammatory factors in zebrafish intestinal tissue after ENR exposure. (K–R) qRT-PCR validation of intestinal barrier- and hypoxia-related genes in zebrafish intestinal tissue after ENR exposure, including *muc2.2* (K), *tjp1a* (L), *tjp1b* (M), *cldn1* (N), *oclna* (O), *oclnb* (P), *hif1aa* (Q), and *hif1ab* (R). Data are presented as mean ± SEM (n = 3 biological replicates per group). Statistical significance was assessed using two-tailed Student’s t-test. **p* < 0.05, ***p* < 0.01, ****p* < 0.001


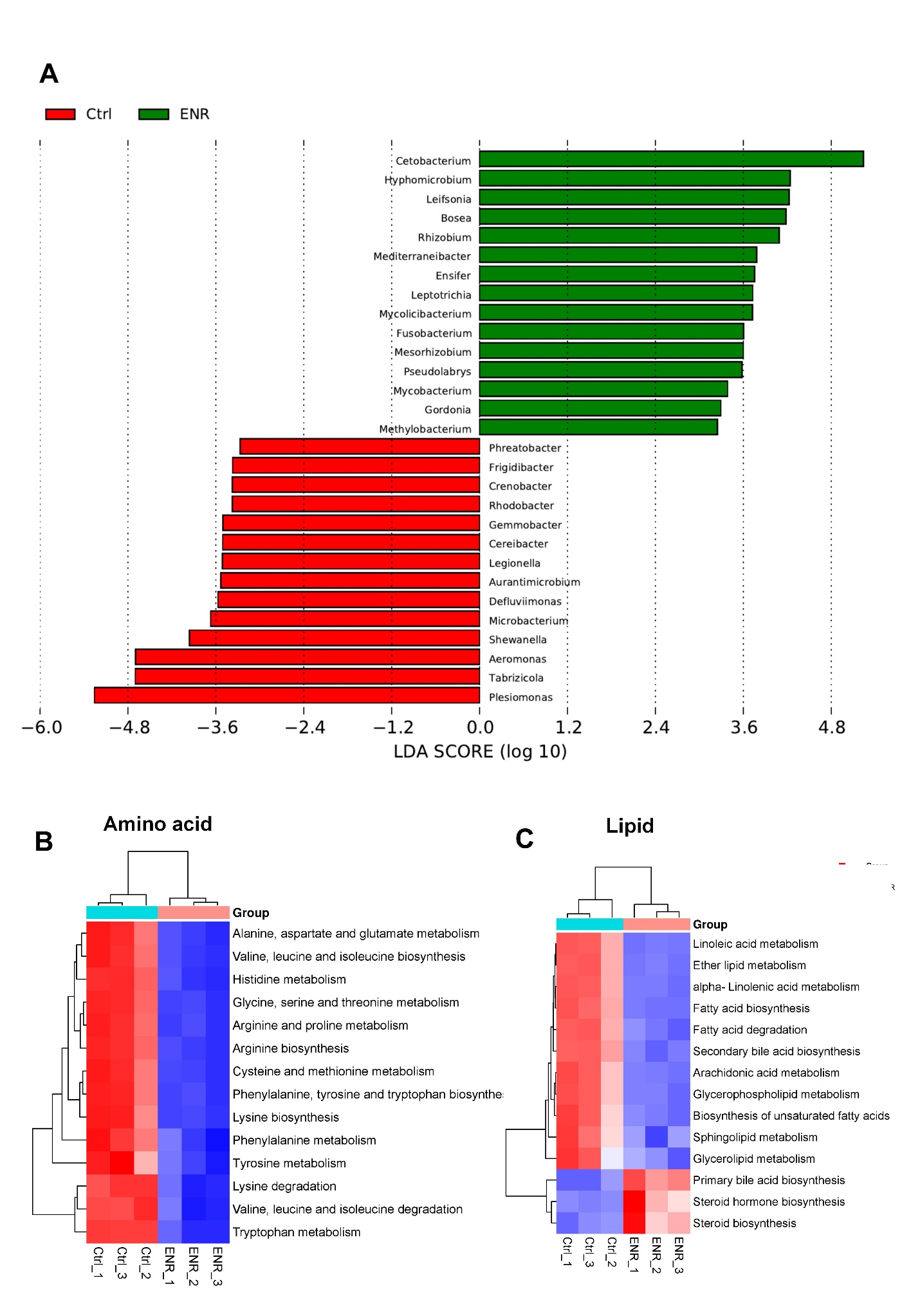


**Supplementary Figure 2.** (A–D) Alpha-diversity index (Ace, Chao, Shannon, Simpson) of gut microbiota at 30 days. (E) LEfSe analysis of the intestinal microbiome at the genus level following 30 days of ENR exposure. (F) Heatmap depicting genes associated with amino acid metabolism in the intestinal microbiome. (G) Heatmap illustrating genes related to lipid metabolism in the intestinal microbiome.


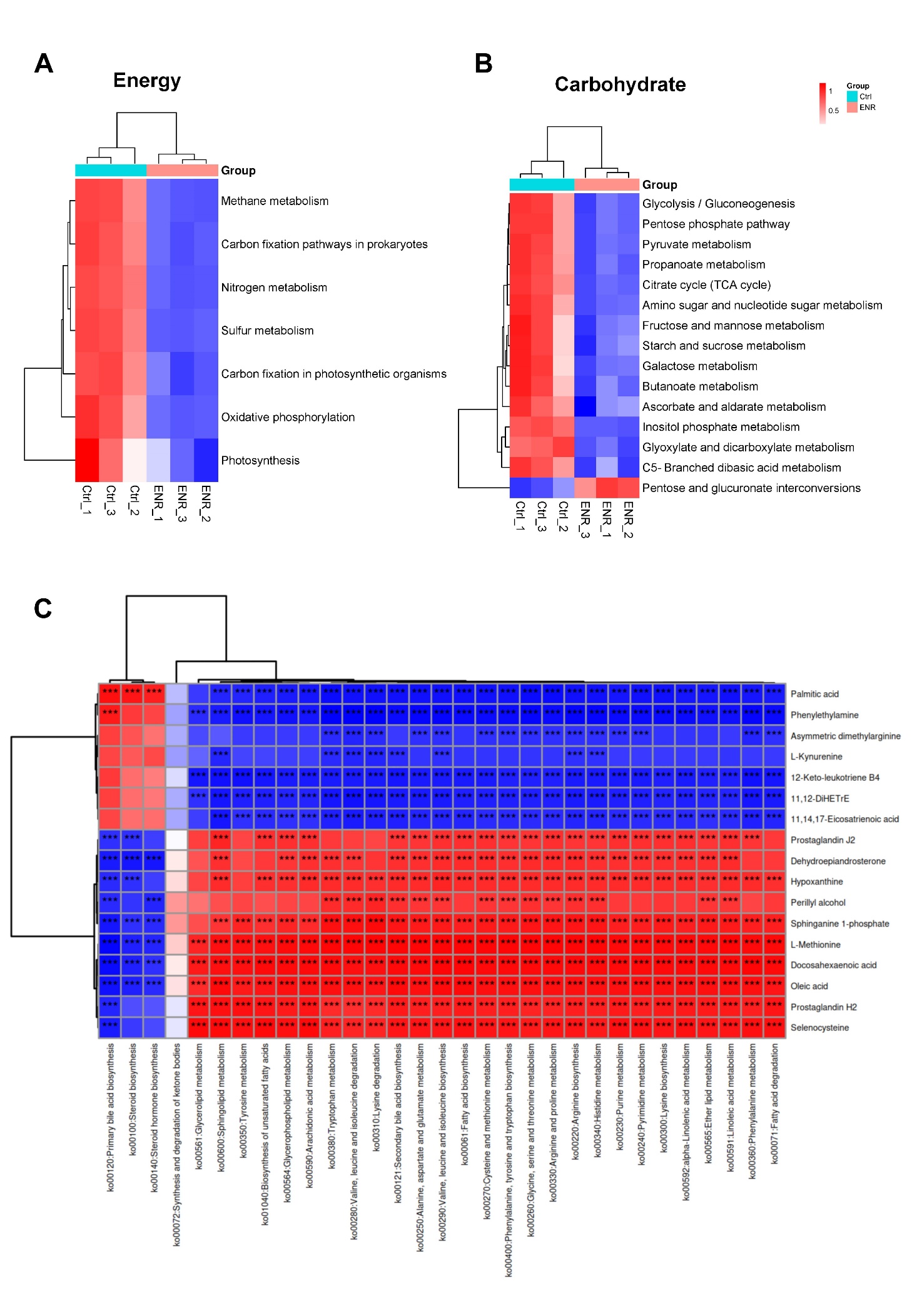


**Supplementary Figure 3. Metagenomic analysis of the gut microbiome following 30 days of ENR exposure.** (A) Heatmap showing genes related to energy metabolism in the gut microbiome. (B) Heatmap displaying genes associated with carbohydrate metabolism in the gut microbiome. (C) Correlation analysis between the abundance of metabolic function-related genes and differential metabolites in the gut.


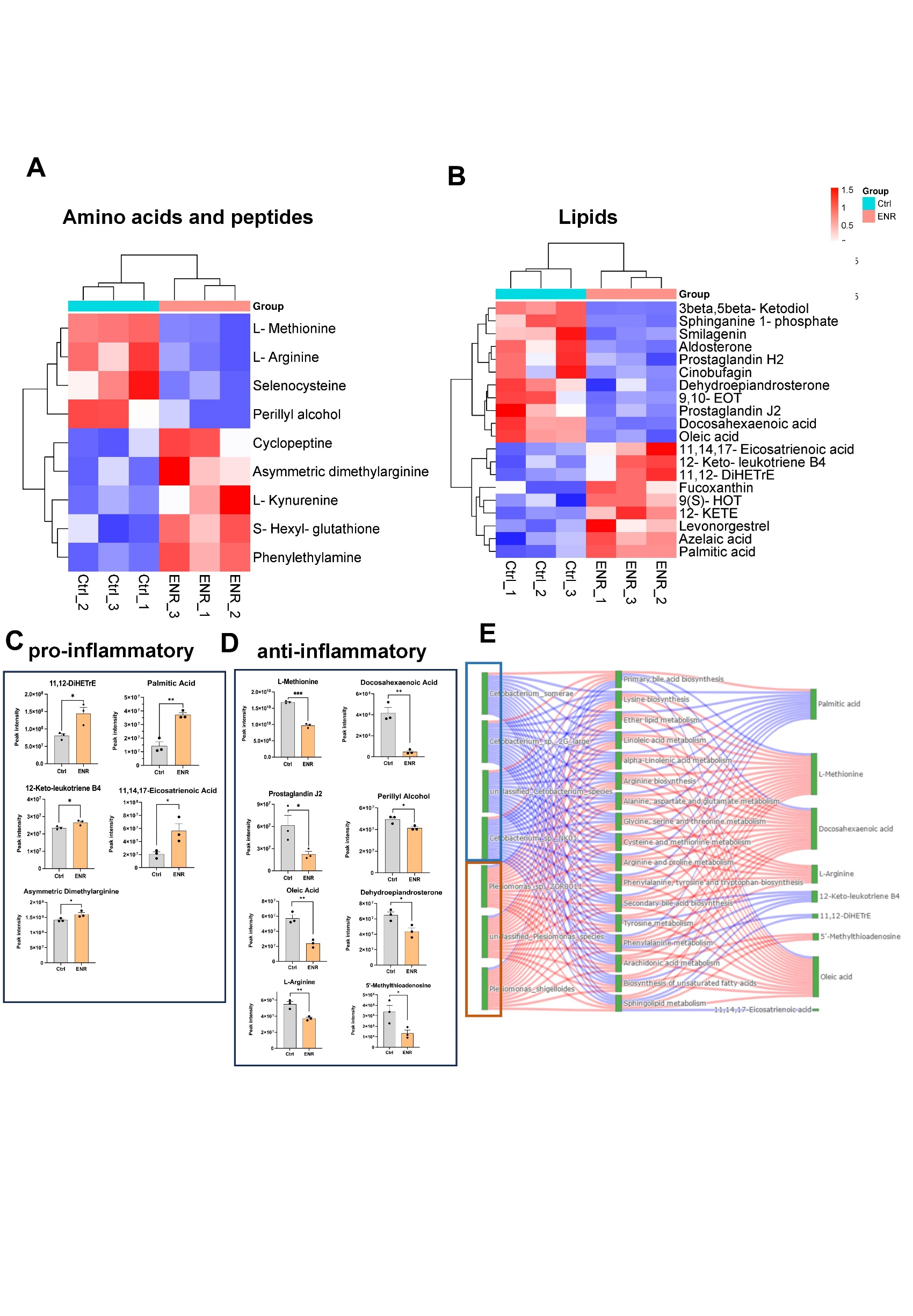


**Supplementary Figure 4. Metabolite analysis of intestinal tissue and its contents.** (A) Amino acid and peptide metabolites. (B) Lipid metabolites. (C) Pro-inflammatory metabolites and (D) anti-inflammatory metabolites. (E) Sankey plot illustrating the relationship between gut microbiota, metabolic function-related genes, and differential metabolites (*p* < 0.05, |*β*| > 0.9). Data are presented as the mean ± standard error of the mean. Statistical significance was assessed using Student's t-test. * *p* < 0.05, ** *p* < 0.01, *** *p* < 0.001.


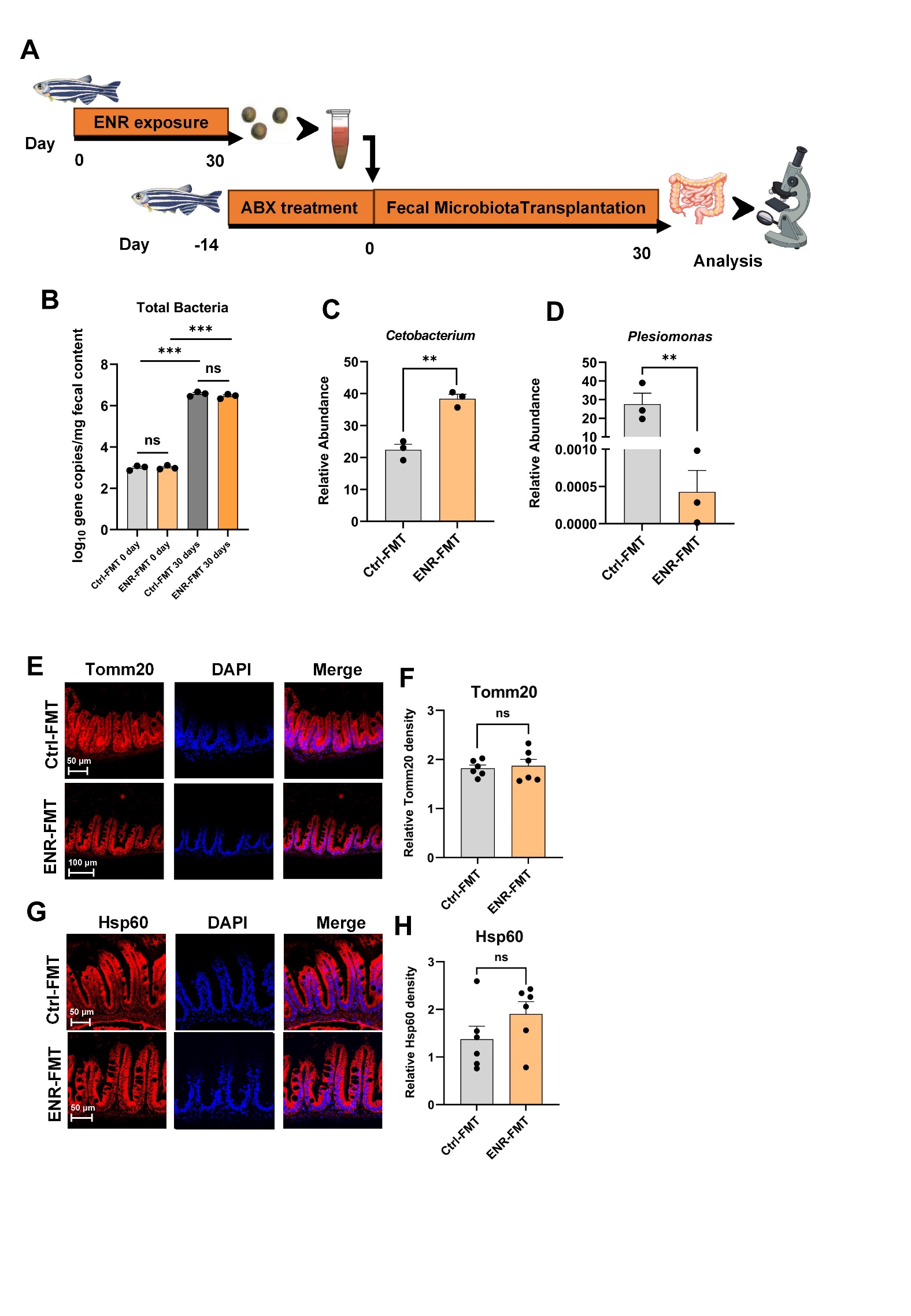


**Supplementary Figure 5.** (A) Flowchart of the fecal microbiota transplantation experiment. (B) Total bacterial load in zebrafish feces was quantified by qRT-PCR. (C-D) Relative abundances of *Cetobacterium* (C) and *Plesiomonas* (D) were measured at day 30 post-FMT. Abundances were normalized to total bacteria 16S rRNA gene levels in each sample. (E-H) Representative immunofluorescence images showing mitochondrial function-related proteins (Tomm20, Hsp60) and their quantification. Data are presented as the mean ± standard error of the mean. Statistical significance was assessed using Student's t-test. * *p* <0.05, ** *p* <0.01, *** *p* <0.001.


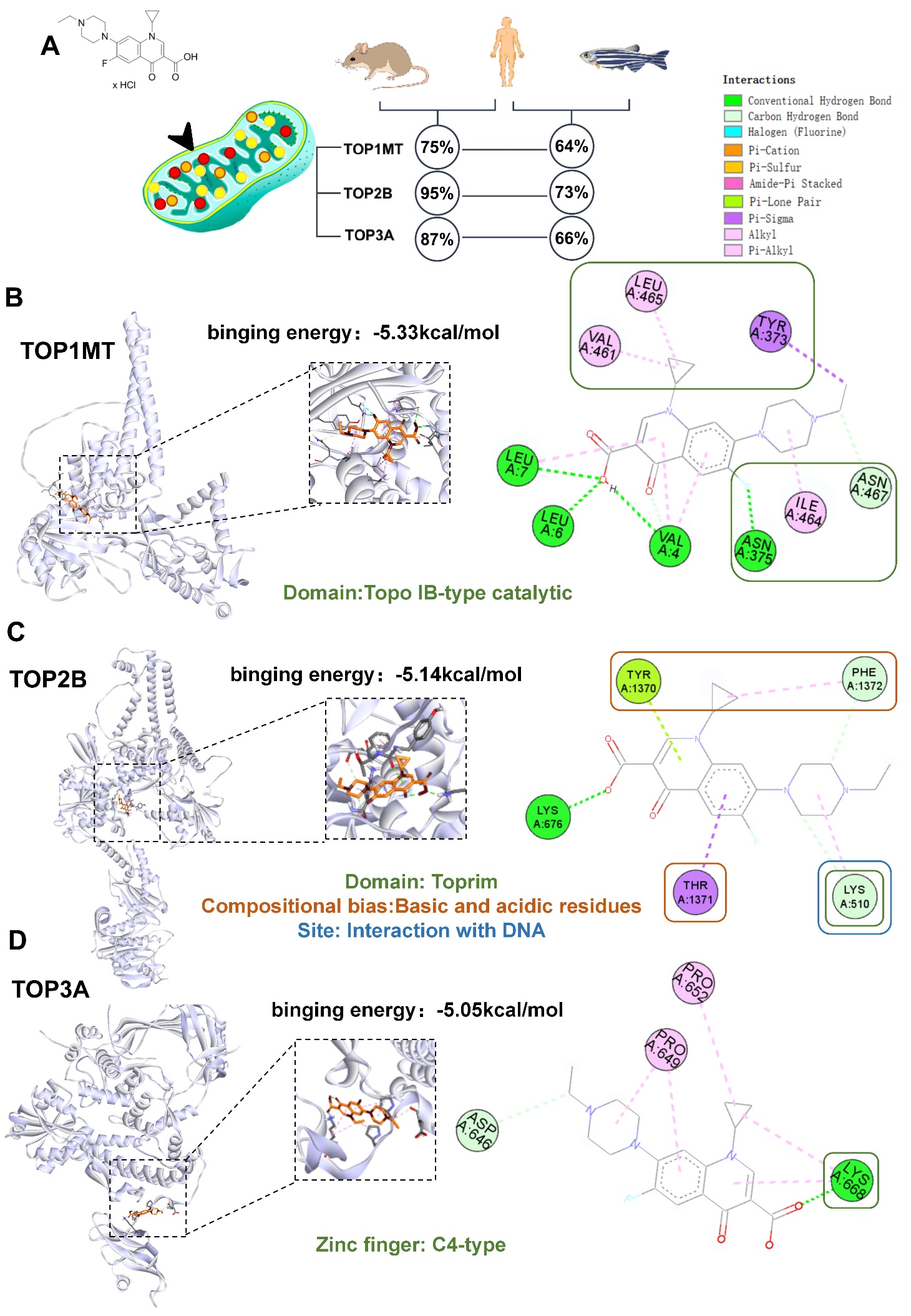


**Supplementary Figure 6.** (A) Homology comparison of topoisomerase proteins between rat, human, and zebrafish. (B) Molecular docking simulation of TOP1MT protein and enrofloxacin (ENR). (C) Molecular docking simulation of TOP2B protein and ENR. (D) Molecular docking simulation of TOP3A protein and ENR.


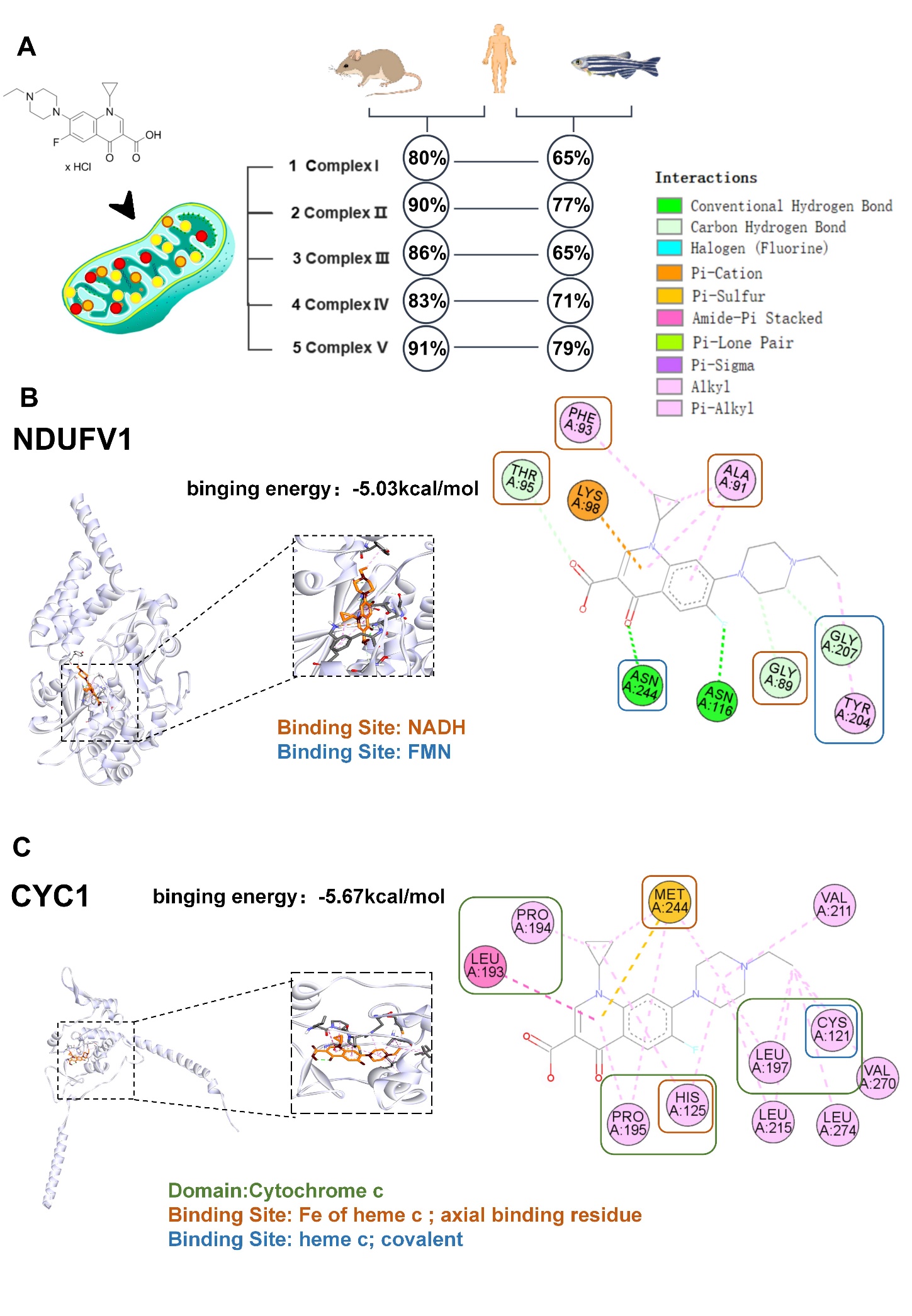


**Supplementary Figure 7.** (A) Homology comparison of mitochondrial complex proteins between rat, human, and zebrafish. (B) Molecular docking simulation of NDUFV1 protein and enrofloxacin (ENR). (C) Molecular docking simulation of CYC1 protein and ENR.


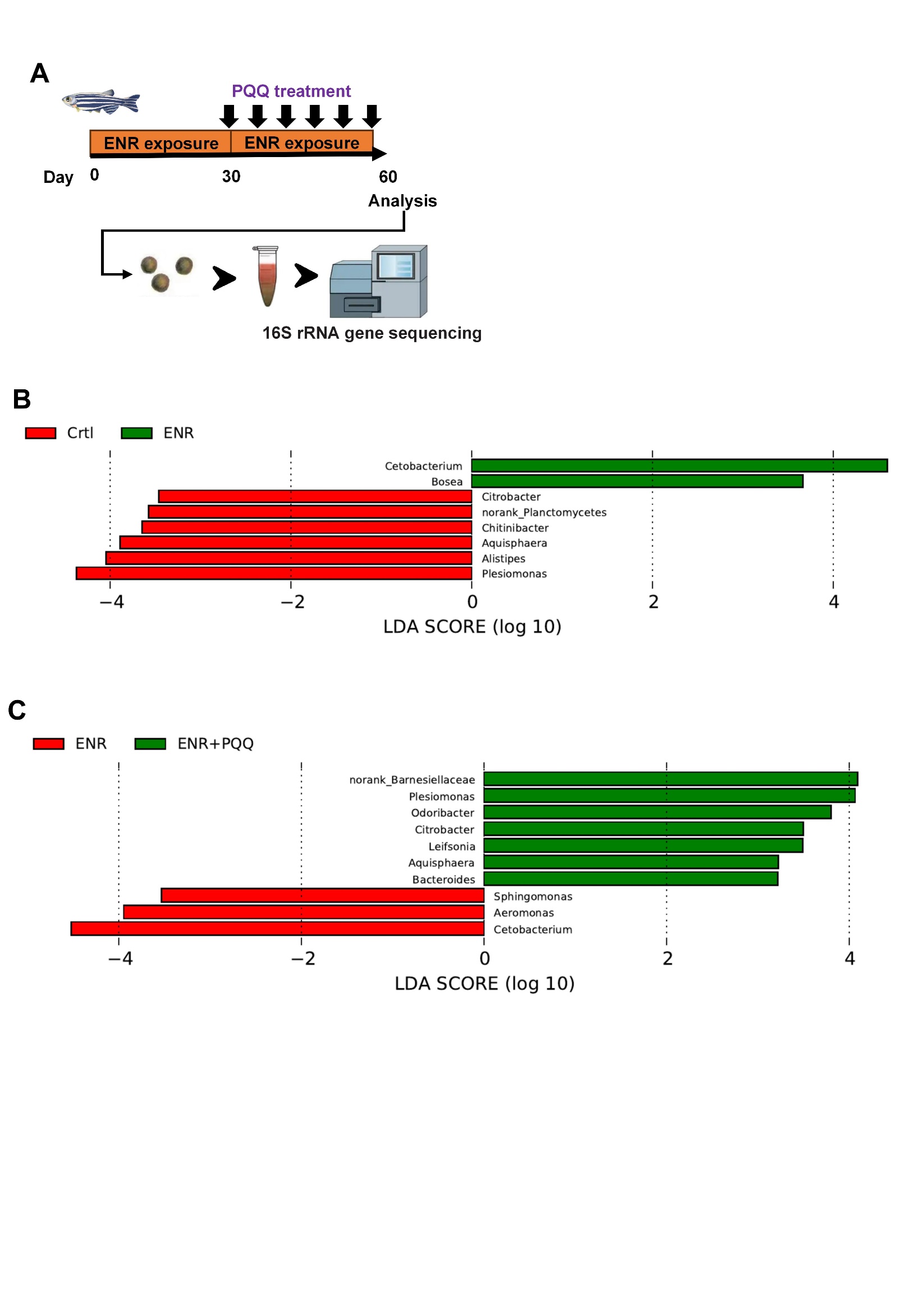


**Supplementary Figure 8.** (A) Experimental flow chart for 16S rRNA gene sequencing and analysis. (B) Genus-level LEfSe analysis comparing the ENR and control groups. (C) Genus-level LEfSe analysis comparing the ENR+PQQ and control groups.


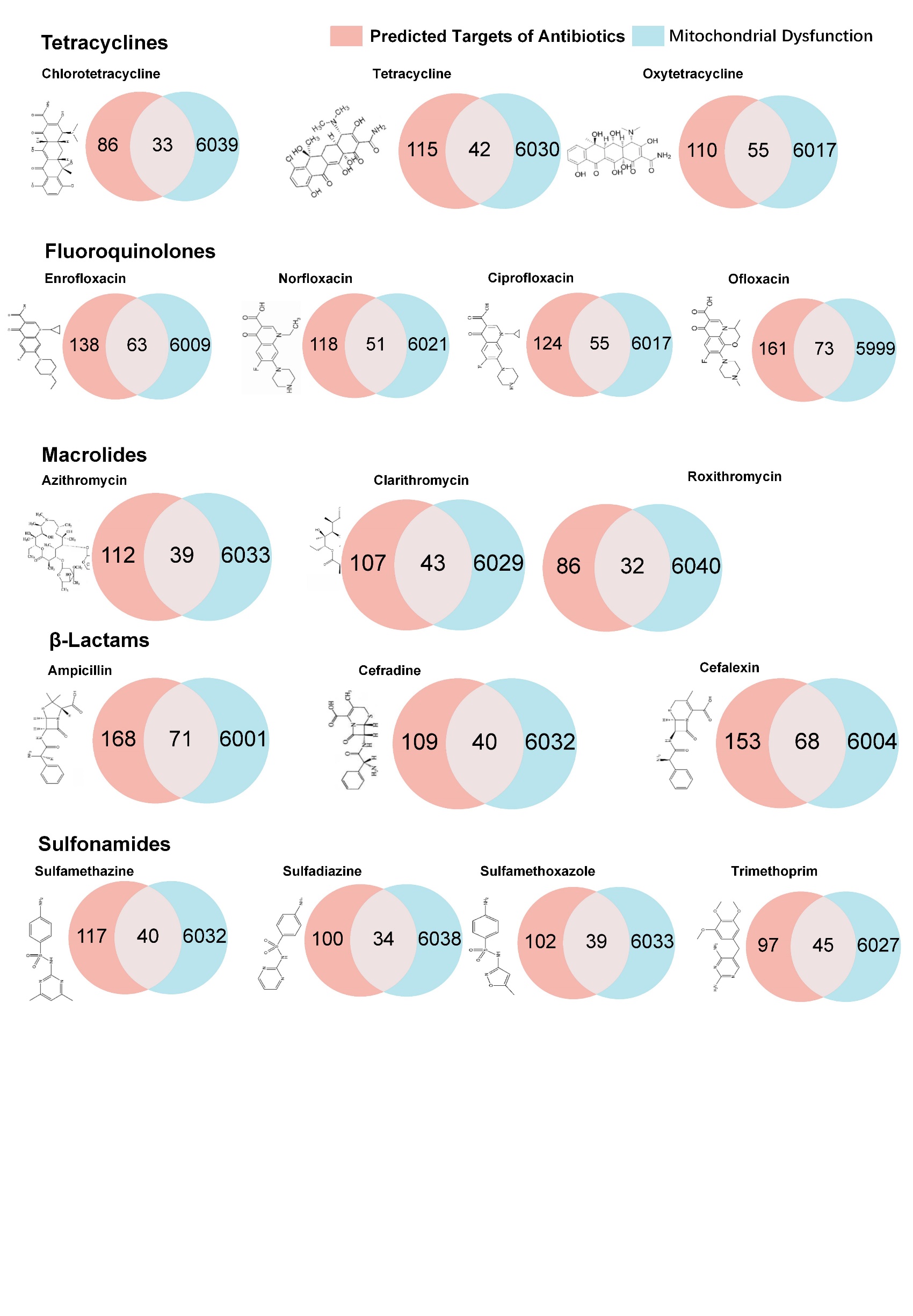


**Supplementary Figure 9. Venn diagram illustrating the overlap among predicted antibiotics targets and Mitochondrial dysfunction-related genes.**


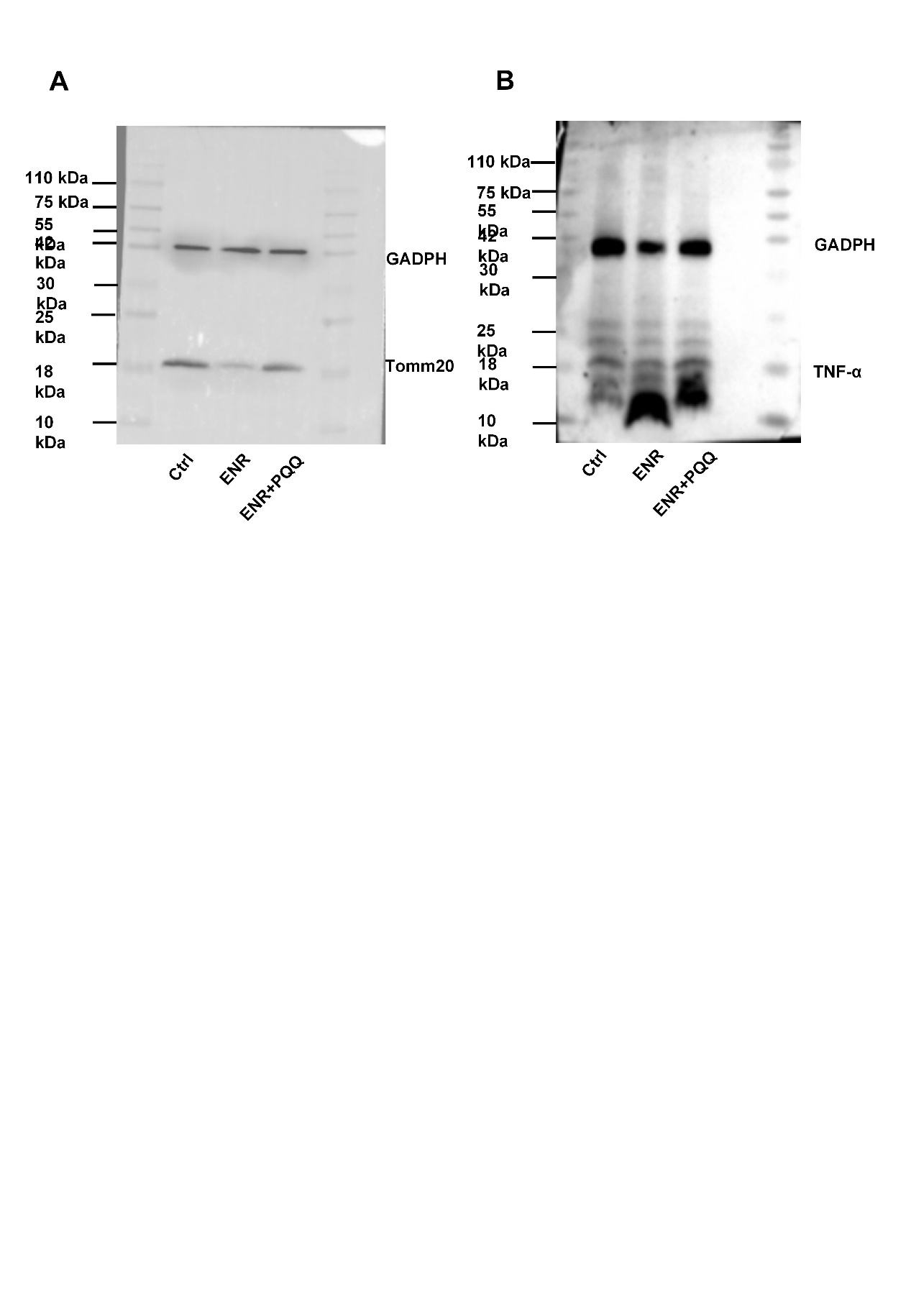


**Supplementary Figure 10.** **Representative Western blot analysis of TOMM20 and TNF-α protein expression.** (A) Whole-membrane Western blot image showing the expression of TOMM20 in three samples. (B) Whole-membrane Western blot image of TNF-α expression.


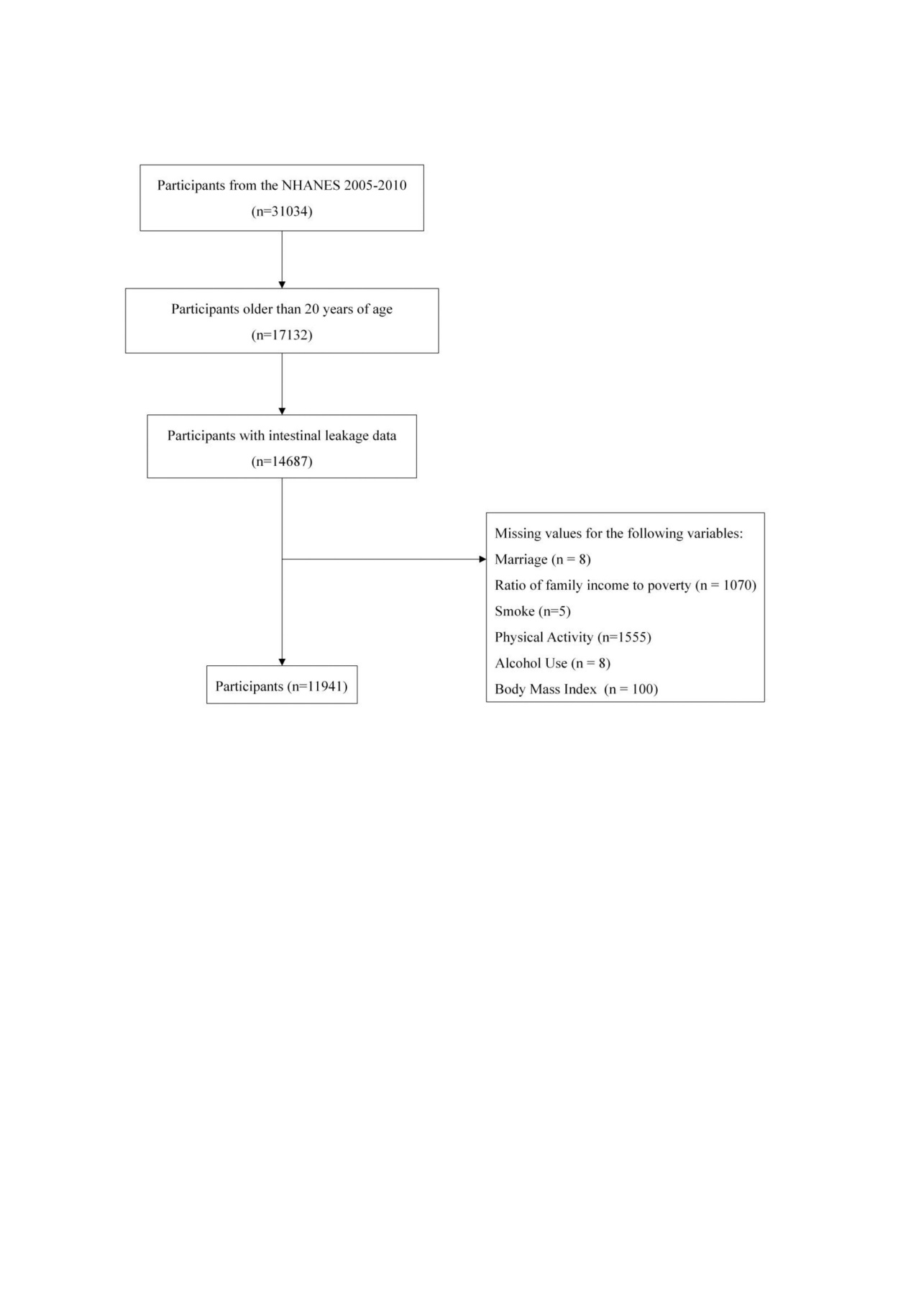


**Supplementary Figure 11. Flowchart of participant inclusion and exclusion in the NHANES analysis.**
