# Supplementary material for: Environmental Enrofloxacin Exposure as a Modifiable Driver of Mitochondria‐Mediated Intestinal Aging and Barrier Dysfunction
Source: Aging Cell. 2026 Apr 30;25(5):e70526. doi: 10.1111/acel.70526 (PMC13130356; doi:10.1111/acel.70526)
Supplement: Supplementary file 2 — Table S1: Baseline characteristics by antibiotic use. Table S2: Stratified analysis of antibiotic use–related KDM and diarrhea by age group. Table S3: DI‐GM and CDAI treatments in relation to antibiotic‐associated KDM and diarrhea in individuals aged 45 and above. Table S4: Components and scoring criteria of DI‐GM in NHANES. Table S5: Primer sequences used for qRT‐PCR in zebrafish and IEC‐6 cells. [file ACEL-25-e70526-s001.docx]

**Table S1. Baseline characteristics by Antibiotic Use**

| **Antibiotic Use mean/proportion** | | | | |
| --- | --- | --- | --- | --- |
| Variables | Total  (n = 11941) | No Antibiotic Use  (n = 11504) | Antibiotic Use  (n = 437) | *P* |
|  |  |  |  |  |
| **Age (year)** | 48.81 ± 17.78 | 48.89 ± 17.77 | 46.76 ± 17.99 | **0.014** |
| **Ratio of family income to poverty** | 2.62 ± 1.63 | 2.62 ± 1.63 | 2.60 ± 1.67 | 0.818 |
| **Energy (kcal)** | 2066.43 ± 875.86 | 2064.88 ± 869.49 | 2106.93 ± 1029.06 | 0.327 |
| **Body Mass Index (kg/m^2^)** | 29.03 ± 6.69 | 29.03 ± 6.67 | 29.06 ± 7.26 | 0.934 |
| **Sex** |  |  |  | **0.002** |
| Male | 5893 (49.35) | 5709 (49.63) | 184 (42.11) |  |
| Female | 6048 (50.65) | 5795 (50.37) | 253 (57.89) |  |
| **Race** |  |  |  | 0.133 |
| Mexican American | 2022 (16.93) | 1965 (17.08) | 57 (13.04) |  |
| Other Hispanic | 1017 (8.52) | 980 (8.52) | 37 (8.47) |  |
| Non-Hispanic White | 6096 (51.05) | 5856 (50.90) | 240 (54.92) |  |
| Non-Hispanic Black | 2319 (19.42) | 2229 (19.38) | 90 (20.59) |  |
| Other Race | 487 (4.08) | 474 (4.12) | 13 (2.97) |  |
| **Smoking** |  |  |  | **0.044** |
| Never | 6332 (53.03) | 6123 (53.22) | 209 (47.83) |  |
| Former | 3027 (25.35) | 2912 (25.31) | 115 (26.32) |  |
| Now | 2582 (21.62) | 2469 (21.46) | 113 (25.86) |  |
| **Drinking** |  |  |  | 0.150 |
| No | 3284 (27.50) | 3177 (27.62) | 107 (24.49) |  |
| Yes | 8657 (72.50) | 8327 (72.38) | 330 (75.51) |  |
| **Physical Activity** |  |  |  | 0.155 |
| Low physical activity | 4639 (38.85) | 4455 (38.73) | 184 (42.11) |  |
| High physical activity | 7302 (61.15) | 7049 (61.27) | 253 (57.89) |  |
| **Marital status** |  |  |  | 0.460 |
| Married | 6393 (53.54) | 6165 (53.59) | 228 (52.17) |  |
| Widowed | 913 (7.65) | 886 (7.70) | 27 (6.18) |  |
| Divorced | 1317 (11.03) | 1271 (11.05) | 46 (10.53) |  |
| Separated | 380 (3.18) | 361 (3.14) | 19 (4.35) |  |
| Never married | 2000 (16.75) | 1923 (16.72) | 77 (17.62) |  |
| Living with partner | 938 (7.86) | 898 (7.81) | 40 (9.15) |  |
| **White blood cell count**  **(1000 cells/uL)** | 7.26 ± 2.57 | 7.25 ± 2.58 | 7.50 ± 2.38 | 0.051 |
| **C-reactive protein(mg/dL)** | 0.43 ± 0.81 | 0.42 ± 0.81 | 0.46 ± 0.74 | 0.417 |
| **DI-GM** | 5.06 ± 1.74 | 5.06 ± 1.74 | 4.98 ± 1.80 | 0.402 |
| **CDAI** | 0.46 ± 3.50 | 0.46 ± 3.50 | 0.39 ± 3.50 | 0.681 |
| **KDM** | 42.89 ± 19.79 | 42.93 ± 19.73 | 41.73 ± 21.14 | 0.222 |
| **KDM-Advance** |  |  |  | **0.035** |
| Deceleration | 7745 (68.12) | 7480 (65.02) | 265 (60.64) |  |
| Acceleration | 3624 (31.88) | 3471 (30.17) | 153 (35.01) |  |
| **Diarrhea** |  |  |  | **0.023** |
| Always | 19 (0.40) | 19 (0.17) | 0 (0.00) |  |
| Most of the time | 62 (1.30) | 60 (0.52) | 2 (0.46) |  |
| Sometimes | 1007 (21.15) | 961 (8.35) | 46 (10.53) |  |
| Rarely | 1921 (40.34) | 1852 (16.10) | 69 (15.79) |  |
| Never | 1751 (36.77) | 1702 (14.79) | 49 (11.21) |  |

Mean ± SD for continuous variables and n (%) for categorical variable.

**Table S2. Stratified Analysis of Antibiotic Use–Related KDM and Diarrhea by Age Group**

| **Antibiotic Use** | | | | | |
| --- | --- | --- | --- | --- | --- |
|  | | Age | OR (95%CI) | *P* | *P* for interaction |
| KDM | Mode1 | 20-85 | 1.09 (0.79 ~ 1.50) | 0.605 |  |
|  |  | 20-45 | 0.77 (0.55 ~ 1.08) | 0.125 | **0.018** |
|  |  | 45-85 | **1.64 (1.01 ~ 2.68)** | **0.047** |  |
|  | Mode 2 | 20-85 | 1.08 (0.79 ~ 1.48) | 0.620 |  |
|  |  | 20-45 | 0.75 (0.54 ~ 1.05) | 0.093 | **0.015** |
|  |  | 45-85 | **1.64 (1.00 ~ 2.67)** | **0.049** |  |
| Diarrhea | Mode 1 | 20-85 | **1.53 (1.04 ~ 2.26)** | **0.048** |  |
|  |  | 20-45 | 0.97 (0.58 ~ 1.63) | 0.920 | 0.108 |
|  |  | 45-85 | **2.66 (1.10 ~ 6.41)** | **0.045** |  |
|  | Mode 2 | 20-85 | **1.57 (1.04 ~ 2.38)** | **0.048** |  |
|  |  | 20-45 | 0.98 (0.59 ~ 1.63) | 0.925 | 0.093 |
|  |  | 45-85 | **2.76 (1.13 ~ 6.77)** | **0.041** |  |

Model 1 is adjusted for age, sex, PIR, BMI, race, smoking, drinking, marital status, and energy intake.

Model 2 is additionally adjusted for white blood cell count and serum CRP based on Model 1.

**Table S3. DI-GM and CDAI Treatments in Relation to Antibiotic-Associated KDM and Diarrhea in Individuals Aged 45 and Above**

| **Antibiotic Use** | | | | | |
| --- | --- | --- | --- | --- | --- |
|  |  | Subgroup | OR (95%CI) | *P* | P for interaction |
| KDM | Mode 1 | DI GM MEAN median |  |  | **0.006** |
|  |  | Low | 3.32 (1.95 ~ 5.64) | **< 0.001** |  |
|  |  | High | 0.91 (0.42 ~ 1.97) | 0.813 |  |
|  |  | CDAI MEAN median |  |  | 0.138 |
|  |  | Low | 2.37 (1.22 ~ 4.62) | **0.015** |  |
|  |  | High | 1.07 (0.50 ~ 2.28) | 0.866 |  |
|  |  | GMCDAI |  |  | 0.138 |
|  |  | Low | 3.76 (1.95 ~ 7.25) | **< 0.001** |  |
|  |  | Middle | 1.45 (0.62 ~ 3.42) | 0.401 |  |
|  |  | High | 0.84 (0.26 ~ 2.66) | 0.763 |  |
|  | Model 2 | DI GM MEAN median |  |  | **0.006** |
|  |  | Low | 3.50 (2.02 ~ 6.08) | **< 0.001** |  |
|  |  | High | 0.92 (0.42 ~ 2.00) | 0.826 |  |
|  |  | CDAI MEAN median |  |  | 0.135 |
|  |  | Low | 2.35 (1.23 ~ 4.49) | **0.013** |  |
|  |  | High | 1.07 (0.51 ~ 2.26) | 0.850 |  |
|  |  | GMCDAI |  |  | 0.102 |
|  |  | Low | 4.47 (2.18 ~ 9.16) | **< 0.001** |  |
|  |  | Middle | 1.42 (0.62 ~ 3.26) | 0.412 |  |
|  |  | High | 0.85 (0.27 ~ 2.67) | 0.781 |  |
| Diarrhea | Mode 1 | DI GM MEAN median |  |  | 0.757 |
|  |  | Low | 3.77 (1.48 ~ 9.64) | **0.006** |  |
|  |  | High | 3.04 (0.69 ~ 13.34) | 0.140 |  |
|  |  | CDAI MEAN median |  |  | 0.837 |
|  |  | Low | 3.60 (1.03 ~ 12.61) | **0.045** |  |
|  |  | High | 3.13 (0.60 ~ 16.41) | 0.177 |  |
|  |  | GMCDAI |  |  | 0.950 |
|  |  | Low | 4.39 (1.07 ~ 17.96) | **0.039** |  |
|  |  | Middle | 3.31 (0.75 ~ 14.66) | 0.115 |  |
|  |  | High | 3.26 (0.50 ~ 21.16) | 0.215 |  |
|  | Model 2 | DI GM MEAN median |  |  | 0.817 |
|  |  | Low | 3.59 (1.43 ~ 9.03) | **0.007** |  |
|  |  | High | 3.09 (0.71 ~ 13.57) | 0.134 |  |
|  |  | CDAI MEAN median |  |  | 0.816 |
|  |  | Low | 3.64 (1.04 ~ 12.74) | **0.043** |  |
|  |  | High | 3.17 (0.59 ~ 17.03) | 0.180 |  |
|  |  | GMCDAI |  |  | 0.958 |
|  |  | Low | 4.05 (0.97 ~ 16.89) | 0.055 |  |
|  |  | Middle | 3.34 (0.75 ~ 14.88) | 0.114 |  |
|  |  | High | 3.27 (0.50 ~ 21.29) | 0.216 |  |

Model 1 is adjusted for age, sex, PIR, BMI, race, smoking, drinking, marital status, and energy intake.

Model 2 is additionally adjusted for white blood cell count and serum CRP based on Model 1.

**Table S4. Components and scoring criteria of DI-GM in NHANES.**

| **Components of DI-GM** | **Food items included in NHANES** | **Scoring criteria** |
| --- | --- | --- |
| **Beneficial to gut microbiota** | Avocados | Score 1 - Consumption≥sex-specific median  Score 0 - Otherwise |
|  | Broccoli |  |
|  | Chickpeas |  |
|  | Coffee |  |
|  | Cranberries |  |
|  | Fermented dairy (including yogurt, cheese, kefir, sour cream, buttermilk) |  |
|  | Fiber |  |
|  | Soybean (including Soy milk, Tofu) |  |
|  | Whole grains |  |
| **Unfavorable to gut microbiota** | Refined grains | Score 0 - Consumption≥sex-specific median  Score 1 - Otherwise |
|  | Processed meat |  |
|  | Red meat |  |
|  | High-fat diet (% energy) | Score 0 - Consumption≥40%  Score 1 - Otherwise |

Abbreviations: DI-GM, dietary index for gut microbiota; NHANES, National Health and Nutrition Examination Survey.

**Table S5. Primer sequences used for qRT-PCR in zebrafish and IEC-6 cells.**

| Gene Name | Forward (5’-3’) | Reverse (5’−3’) |
| --- | --- | --- |
| *Gapdh* (Rat) | GCAAGTTCAACGGCACAG | CGCCAGTAGACTCCACGAC |
| *Il-6* (Rat) | TGGAGTTCCGTTTCTACCTG | TTCATATTGCCAGTTCTTCG |
| *Tnf-α* (Rat) | ACCTTATCTACTCCCAGGTTCT | GGCTGACTTTCTCCTGGTATG |
| *β-actin* (Zebrafish) | GTGCCCATCTACGAGGGTTA | TCTCAGCTGTGGTGGTGAAG |
| *tnf-α* (Zebrafish) | GCGCTTTTCTGAATCCTAC | TGCCCAGTCTGTCTCCTTCT |
| *il-6* (Zebrafish) | ACATGACGGCATTTGAAGGG | TATGGCCTCCAGCAGTCGTT |
| *ifn-γ* (Zebrafish) | CATGCAGAATGACAGCGTGG | TTGATGCTTTAGCCTGCCGT |
| *cox-2* (Zebrafish) | AATTTGCTGTGGGCCATGAG | AAGTTGTCCGGCAACAGTGG |
| *ifn-α* (Zebrafish) | GGTGGAATATCTGCAGGTTC | GCTTGAAGCAATGACACCA |
| *cxcr1* (Zebrafish) | CGTTTGTTCCCGACGAGAAG | CAGCGGATGCCATTGTGAT |
| *mt-nd1* (Zebrafish) | TACAGAGGGGGAATCAGAAC | TTGGTCGTATCGGAATCGT |
| *mt-nd2* (Zebrafish) | GACCTACCAGCCACAGCTAC | GGGTCGTTTGTACCCGTCAG |
| *mt-nd4* (Zebrafish) | TAATCGCCCACGGACTAACC | ATGGTCCGGCTATGAGTTCG |
| *mt-nd5* (Zebrafish) | TCCCATCTTAACGCCTGAGC | CGGGGGCTTCCTAAACAGAC |
| *mt-nd6* (Zebrafish) | GTTGTAATTGTGGCGGCTGG | TTGCAACCCCGCTTACATCA |
| *mt-nd4l* (Zebrafish) | ACACCCACGCACTTTAGTCT | GCTTCACAGGCAGAAAAGGC |
| *mt-atp6* (Zebrafish) | TTATCCTCGTTGCCATACTTC | AGTTGGTTTGTGAATCGTCC |
| *mt-atp8* (Zebrafish) | CTCAGCTTAATCCAAAACCCTG | ACTTGAGTTGGGTCATTAGGTTG |
| *mt-co2* (Zebrafish) | AGTCCTTCCGGCCATCATTC | CTGGTGTGAGGTCTTGGGTT |
| *mt-cyb* (Zebrafish) | TTTGTGGGCTACGTCCTTCC | TGTTGGGGTTTAGGCCAAGG |
| *mrps24* (Zebrafish) | GGCTCGTATTCGTGTGGGTAA | CGGGAGTGTCAGCAGGTATG |
| *muc2.2* (Zebrafish) | ACACGCTCAAGTAATCGCACAGTC | TCAGCGAGTGTTTGGCTCACTT |
| *oclna* (Zebrafish) | CAGAATGTCGTCGAAGCACATC | ATGGACAGCACACACATGATCT |
| *oclnb* (Zebrafish) | TGACAAAGTCAGTGCTGCTCA | GGATATTCATCTGTGTCAAGCTCAT |
| *tjp1a* (Zebrafish) | CAAAGACCAACAGCACTGCC | GTGGTTTAGCGGTGATGGGA |
| *tjp1b* (Zebrafish) | GCGAGCAGCTAAAGGAACAAAA | CGTAAGCGTGATCCACATTGTC |
| *cldn1* (Zebrafish) | CTGCTGTATCTGTGGGAGTGAA | TAATCAGGAGAACAGGCGAAG |
| *hif1aa* (Zebrafish) | TGTCAACTACATCCTCAGTGGC | CCTCCTCTTTCAACTCTGCCAT |
| *hif1ab* (Zebrafish) | GAGCCAGATGACCTCTTGAACA | TGGGGTTGTAGATTACAGTGGC |
| 16s | CCTACGGGNGGCWGCAG | GACTACHVGGGTATCTAATCC |
| *Cetobacterium* (genus) | AGTTTGATCCTGGCTCAGGATG | GAGGCAAGTTCCTTACGCGTT |
| *Plesiomonas* (genus) | CTCCGAATACCGTAGAGTGCTATCC | CTCCCCTAGCCCAATAACACCTAAA |
| Total bacteria | CCTACGGGAGGCAGCAG | ATTACCGCGGCTGCTGG |
